# A bispecific antibody targeting sclerostin and DKK-1 promotes bone mass accrual and fracture repair

**DOI:** 10.1038/ncomms11505

**Published:** 2016-05-27

**Authors:** Monica Florio, Kannan Gunasekaran, Marina Stolina, Xiaodong Li, Ling Liu, Barbara Tipton, Hossein Salimi-Moosavi, Franklin J. Asuncion, Chaoyang Li, Banghua Sun, Hong Lin Tan, Li Zhang, Chun-Ya Han, Ryan Case, Amy N. Duguay, Mario Grisanti, Jennitte Stevens, James K. Pretorius, Efrain Pacheco, Heidi Jones, Qing Chen, Brian D. Soriano, Jie Wen, Brenda Heron, Frederick W. Jacobsen, Emil Brisan, William G. Richards, Hua Zhu Ke, Michael S. Ominsky

**Affiliations:** 1 Cardiometabolic Disorders, Amgen Inc., One Amgen Center Drive, Thousand Oaks, California 91320, USA; 2 Therapeutic Discovery, Amgen Inc., One Amgen Center Drive, Thousand Oaks, California 91320, USA; 3 Pharmacokinetics & Drug Metabolism, Amgen Inc., One Amgen Center Drive, Thousand Oaks, California 91320, USA; 4 Comparative Biology & Safety Sciences, Amgen Inc., One Amgen Center Drive, Thousand Oaks, California 91320, USA; 5 Process Development, Amgen Inc., One Amgen Center Drive, Thousand Oaks, California 91320, USA

## Abstract

Inhibition of the Wnt antagonist sclerostin increases bone mass in patients with osteoporosis and in preclinical animal models. Here we show increased levels of the Wnt antagonist Dickkopf-1 (DKK-1) in animals treated with sclerostin antibody, suggesting a negative feedback mechanism that limits Wnt-driven bone formation. To test our hypothesis that co-inhibition of both factors further increases bone mass, we engineer a first-in-class bispecific antibody with single residue pair mutations in the Fab region to promote efficient and stable cognate light–heavy chain pairing. We demonstrate that dual inhibition of sclerostin and DKK-1 leads to synergistic bone formation in rodents and non-human primates. Furthermore, by targeting distinct facets of fracture healing, the bispecific antibody shows superior bone repair activity compared with monotherapies. This work supports the potential of this agent both for treatment and prevention of fractures and offers a promising therapeutic approach to reduce the burden of low bone mass disorders.

## Abstract (web summary)

Antibodies that block the Wnt inhibitors sclerostin and DKK- 1 enhance bone formation and fracture repair. Here the authors show these monospecific antibodies induce compensatory mechanisms that limit efficacy, and have designed a sclerostin/DKK-1 bispecific antibody that promotes superior fracture repair in rodents and bone formation in primates.

## Graphical abstract

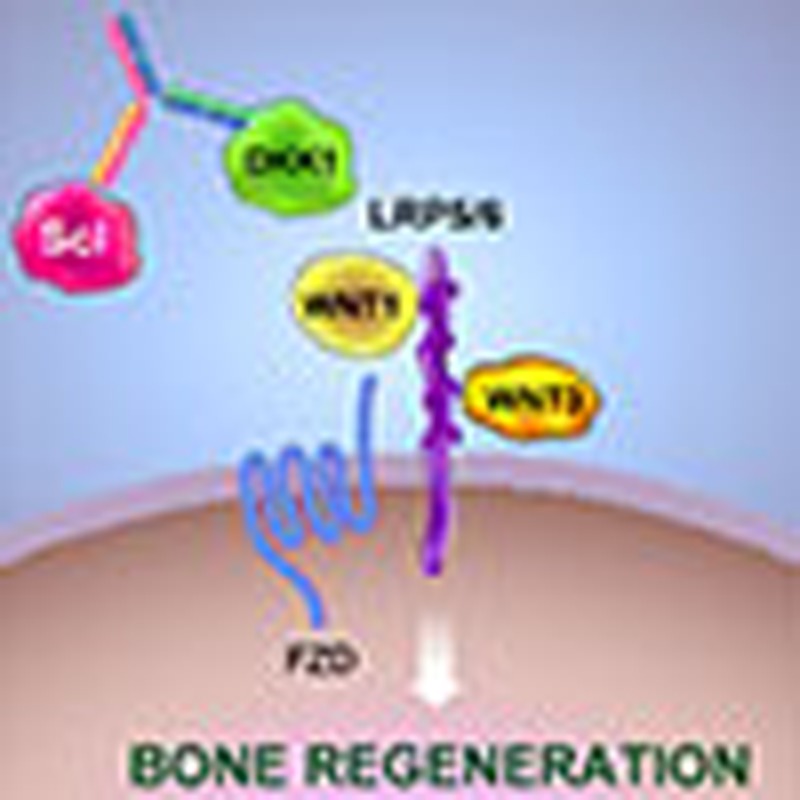

Wnt signalling provides critical cues to promote osteoblastogenesis and bone formation that occur during growth, bone homoeostasis or fracture repair. A number of extracellular Wnt antagonists regulate bone formation by binding directly to Wnt ligands or by competing with Wnt ligands for binding to the co-receptors lipoprotein-related proteins 5 and 6 (LRP5 and LRP6) expressed on the surface of bone cells^1^. Sclerostin is a secreted factor produced by osteocytes that blocks Wnt signalling at least in part by binding to LRP5 and LRP6 (refs 2, 3). Genetic deletion of sclerostin results in high bone mass due to increased bone formation in mice and humans^4^,^5^,^6^. Dickkopf-1 (DKK-1) is another secreted Wnt antagonist that blocks binding of Wnt proteins to LRP5 and LRP6, although it does so by binding a larger region on the receptor's extracellular surface and thereby blocks additional classes of Wnt proteins^7^,^8^,^9^,^10^. The deletion of *DKK1* in mice results in postnatal lethality and severe developmental phenotypes including head defects and limb dysmorphogenesis^11^. Mutations in *LRP5* that lead to high bone mass phenotypes in rodents and humans decrease binding to both sclerostin and DKK-1 (refs 12, 13, 14). In a meta-analysis of 17 genome-wide association studies, both *SOST* and *DKK1* variants were associated with bone mineral density (BMD) and fracture risk^15^, suggesting an association with osteoporosis.

Antibodies that neutralize sclerostin (Scl-Ab) or DKK-1 (DKK1-Ab) are being evaluated as potential therapies to treat bone disorders such as post-menopausal osteoporosis and myeloma-induced bone disease^16^,^17^,^18^,^19^,^20^,^21^. The bone-forming potential of Scl-Ab has been demonstrated previously^16^,^22^. Smaller increments in BMD occurred in preclinical species after administration of DKK1-Ab^23^. Other data show that Scl-Ab and DKK1-Ab improve fracture healing in animal models, effects associated with increased bone formation^23^,^24^. Furthermore, the involvement of DKK-1 in fracture repair is suggested by a study demonstrating that DKK-1 expression is elevated in fracture tissue of patients with nonunion^25^.

On the basis of mechanistic aspects of DKK-1 and sclerostin interactions with LRP receptors defined by *in vitro* and crystallography studies^7^,^8^,^9^, as well as mouse and human genetics, these proteins probably have distinct and redundant roles in bone formation and repair. Here we show that sclerostin inhibition or *SOST* deficiency leads to a compensatory increase in DKK-1 expression. Therefore, we hypothesize that blocking both proteins further increases Wnt signalling, resulting in a more robust effect on bone formation and repair. The synergistic bone-forming effects of combined Scl-Ab and DKK1-Ab administration in intact as well as disease and injury models provide the basis for engineering a bispecific heterodimeric antibody (Hetero-DS) that inhibits both molecules. Herein, we demonstrate that Hetero-DS has attractive manufacturability attributes and leads to increases in bone formation and repair that are superior to the effects of administration of parental monospecific antibodies.

## Results

### Inhibiting Scl and DKK-1 promotes synergistic bone formation

**Figure 1:**
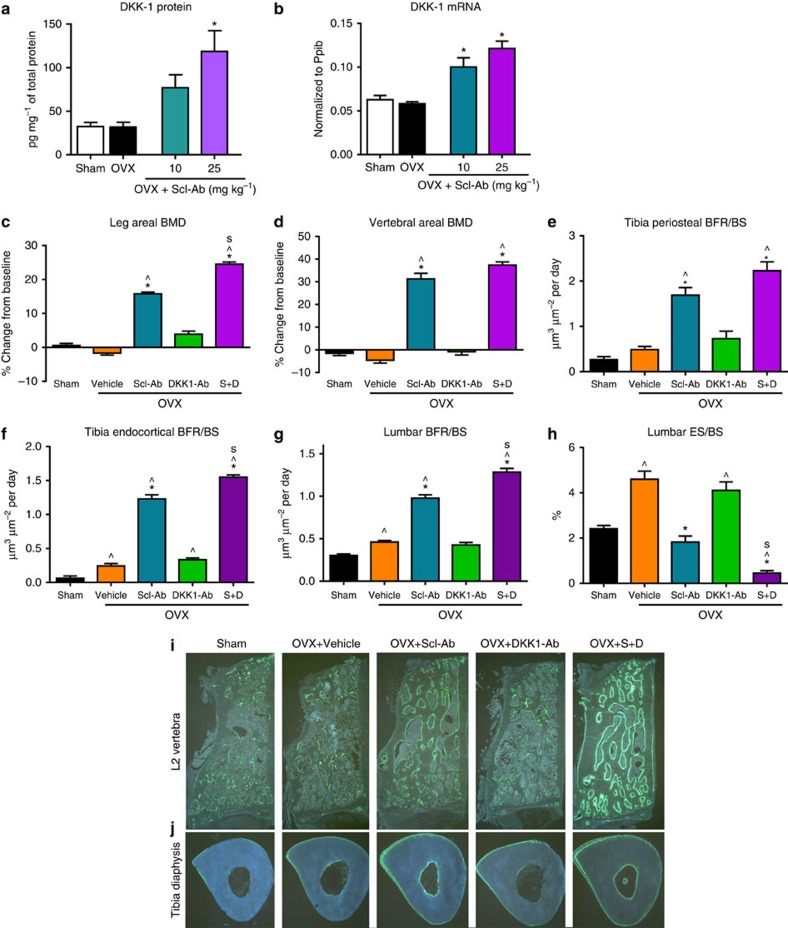
Dual inhibition of DKK-1 and sclerostin led to synergistic bone formation in ovariectomized rats. In 11-month-old ovariectomized (OVX) rats, treatment with sclerostin antibody (Scl-Ab) for 5 weeks led to significant increases in whole bone tissue DKK-1 protein and mRNA levels (*n*=10 per group) (**a**,**b**). Scl-Ab+DKK-1 antibody (DKK1-Ab) robustly increased bone formation and bone mass in OVX rats. Eight-month-old Sprague-Dawley rats underwent ovariectomy and 2 months later were injected subcutaneously twice weekly with Vehicle, Scl-Ab (18.2 mg kg^−1^), DKK1-Ab (18.2 mg kg^−1^) or Scl-Ab+DKK1-Ab (S+D; 18.2 mg kg^−1^ each) for 5 weeks, with a sham-operated group-administered vehicle (*n*=10/group). DXA areal BMD at the (**c**) leg and (**d**) L1–L5 vertebrae expressed as a percentage change from baseline. Histomorphometry reflected BFR/BS at the tibial diaphysis on (**e**) periosteal and (**f**) endocortical surfaces, and on (**g**) trabecular surfaces at the second lumbar vertebra, as well as the bone resorption parameter (**h**) ES/BS. Representative fluorescent micrographs from the (**i**) L2 vertebra and (**j**) tibia diaphysis reflecting bone formation via calcein labels injected 13 and 3 days before termination. Data represent one experiment with 10 rats per group and are presented as mean±s.e.m.; *P<0.05 versus OVX-Vehicle, ^*P*<0.05 versus Sham-vehicle, ^s^*P*<0.05 versus Scl-Ab by analysis of variance+Tukey's post hoc test.

In previous clinical and preclinical studies we've shown that increases in bone formation markers wane over time following sclerostin antibody administration^16^,^26^. A negative feedback loop is further suggested by a study showing *DKK1* is a direct transcriptional target of β-catenin^27^. We hypothesized that DKK-1 may be elevated after sclerostin inhibition in response to Wnt pathway activation. To test our hypothesis, we measured DKK-1 expression in whole-bone lysate in SOST knockout mice and in mature ovariectomized (OVX) rats after Scl-Ab treatment^23^ and found *DKK1* mRNA and protein were upregulated. (Fig. 1a,b and Supplementary Fig. 1). These results suggest that increased *DKK1* gene and protein expression in rodent bone tissue is a result of sclerostin inhibition and Wnt pathway activation *in vivo*. Furthermore, this observation raised the possibility that elevated DKK-1 may limit the effect of sclerostin inhibition on bone mass accrual as part of a negative feedback mechanism. To determine if simultaneous inhibition of sclerostin and DKK-1 could further increase bone formation and bone mass, OVX rats were dosed subcutaneously twice weekly with vehicle, Scl-Ab (25 mg kg^−1^), DKK1-Ab (25 mg kg^−1^), or both of these antibodies (S+D) for 5 weeks. Combination therapy resulted in the largest increases from baseline in areal BMD by dual-energy X-ray absorptiometry (DXA) analysis in the leg and lumbar spine compared with vehicle- or monotherapy-treated rats (Fig. 1c,d). These BMD increases were associated with robust increases in bone formation rate (BFR/BS) on the periosteal and endocortical surfaces of the tibial diaphysis and on cancellous surfaces in lumbar vertebra (Fig. 1e–j and Supplementary Table 1). At the lumbar vertebra, S+D also significantly reduced eroded surface (ES/BS) relative to the vehicle or monotherapy groups (Fig. 1h). DKK1-Ab alone did not significantly affect any of these parameters. Therefore, these results demonstrate a synergistic effect of inhibiting both sclerostin and DKK-1 relative to either Wnt antagonist alone.

**Figure 2:**
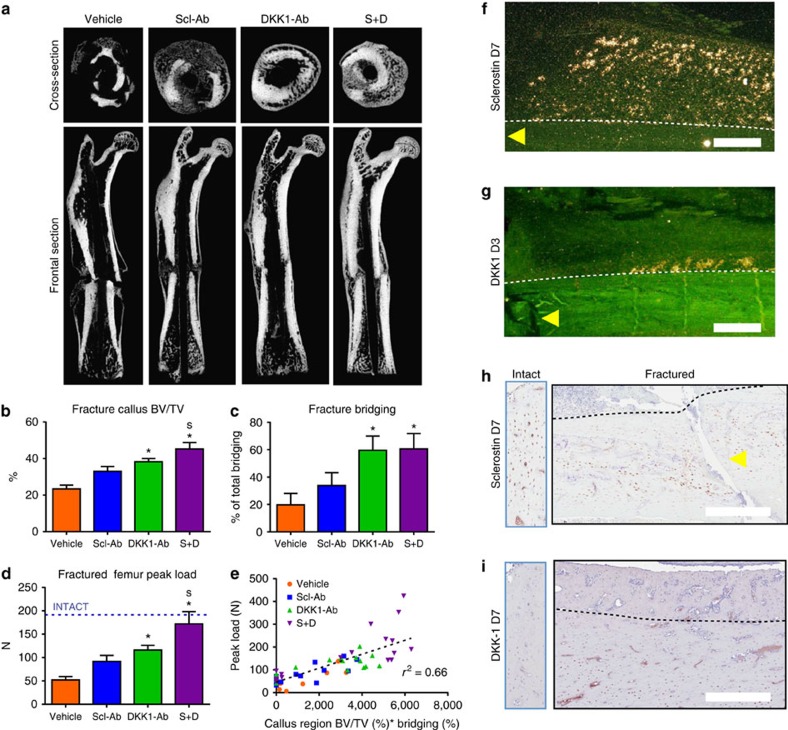
Sclerostin antibody (Scl-Ab)+DKK-1 antibody (DKK1-Ab) improved fracture repair in a rat closed femur fracture model. Seven-month-old male Sprague-Dawley rats underwent closed femur fracture surgery and were injected subcutaneously twice weekly with vehicle, Scl-Ab (25 mg kg^−1^), DKK1-Ab (25 mg kg^−1^) or Scl-Ab+DKK1-Ab (S+D; 25 mg kg^−1^ each) for 7 weeks (*n*=18 for vehicle, *n*=14 for Scl-Ab, *n*=17 for Dkk1-Ab, *n*=17 for S+D). (**a**) Representative transverse and frontal images of the fractured femur by microCT. MicroCT parameters included (**b**) callus BV per TV and (**c**) percentage of total bridging. (**d**) Callus strength (peak load) as measured by three-point bending; dashed line represents the vehicle group mean for intact femur peak load. (**e**) Peak load of healed femurs was correlated with callus BV/TV and % bridging by multiple regression analysis. *In situ* hybridization demonstrated expression of (**f**) SOST and (**g**) DKK-1 in early callus osteocytes at day 7 and in the periosteum at day 3, respectively. Immunohistochemistry demonstrated presence of (**h**) sclerostin and (**i**) DKK-1 protein in cortical osteocytes near and distal to the fracture, respectively, at day 7 (left panels—intact bone). For **f**,**g**, yellow arrows indicate the location of the fracture line, dashed lines outline the original cortex and white bars indicate scale of 500 μm (additional images are provided in Supplemental Fig. 2). Data are from one experiment with 14–18 rats per group and are presented as mean±s.e.m. *P<0.05 versus vehicle, ^s^*P*<0.05 versus Scl-Ab by ANOVA+Tukey's post hoc test. TV, total volume.

To determine whether co-inhibiting sclerostin and DKK-1 could similarly improve bone healing, 7-month-old male rats underwent femoral closed fracture followed by treatment with vehicle, Scl-Ab, DKK1-Ab or S+D for 7 weeks. Callus bone volume (BV) was highest in the S+D group and fracture bridging was improved in both groups receiving DKK1-Ab (Fig. 2a–c). These improvements in the S+D group were associated with the greatest functional improvements in callus bending strength, such that peak load was within 10% of the intact femur vehicle control mean (Fig. 2d). Regression analysis showed a strong correlation between BV, bridging and callus strength (Fig. 2e).

The spatial and temporal expression of sclerostin and DKK-1 were evaluated in the rat closed femur fracture model by *in situ* hybridization (ISH) and immunohistochemistry (IHC) at timepoints up to 42 days post fracture. Both *SOST* and *DKK1* expression increased in maturing osteocytes of the external callus after day 7, with DKK-1 induction also observed on day 3 in the periosteal region adjacent to the fracture line (Fig. 2f,g). Sclerostin protein, evident in the majority of osteocytes in intact bone, was more heterogeneously expressed in osteocytes near the fracture line within the first 14 days after fracture (Fig. 2h and Supplementary Fig. 2a–c). DKK-1 protein, not evident by IHC in the intact rat femurs, was increased in cortical osteocytes in a region distal from the immediate fracture line by day 7, returning towards intact levels as bony bridging was established at week 5 (Fig. 2i and Supplementary Fig. 2d–f). Similar to the ISH results, sclerostin and DKK-1 protein were evident in osteocytes of the maturing callus after day 14. Consistent staining was not observed in the fibrous or cartilaginous callus by either method.

### Engineering a bispecific IgG against DKK-1 and sclerostin

Clinical development of two unapproved agents for use in combination therapy poses significant challenges due to high costs and the length of clinical studies which first have to demonstrate safety and efficacy for each monotherapy and then for the combination of the two^28^. Given the robust effect of combination therapy in our rodent bone studies, we engineered a human bispecific heterodimeric antibody directed against both ligands. This immunoglobulin G (IgG)-like protein was created from two different heavy chains and two different light chains. To drive specific pairing of the appropriate light and heavy chains, charged residue pair mutations (CPMs) were introduced at the heavy-heavy chain and the light-heavy chain interface to discourage undesired homodimers and light-heavy chain mispairing, while promoting desired heavy-chain heterodimerization and cognate light-heavy pairing. The resulting bispecific antibody is an IgG-like molecule without linkers or domain fusions.

**Figure 3:**
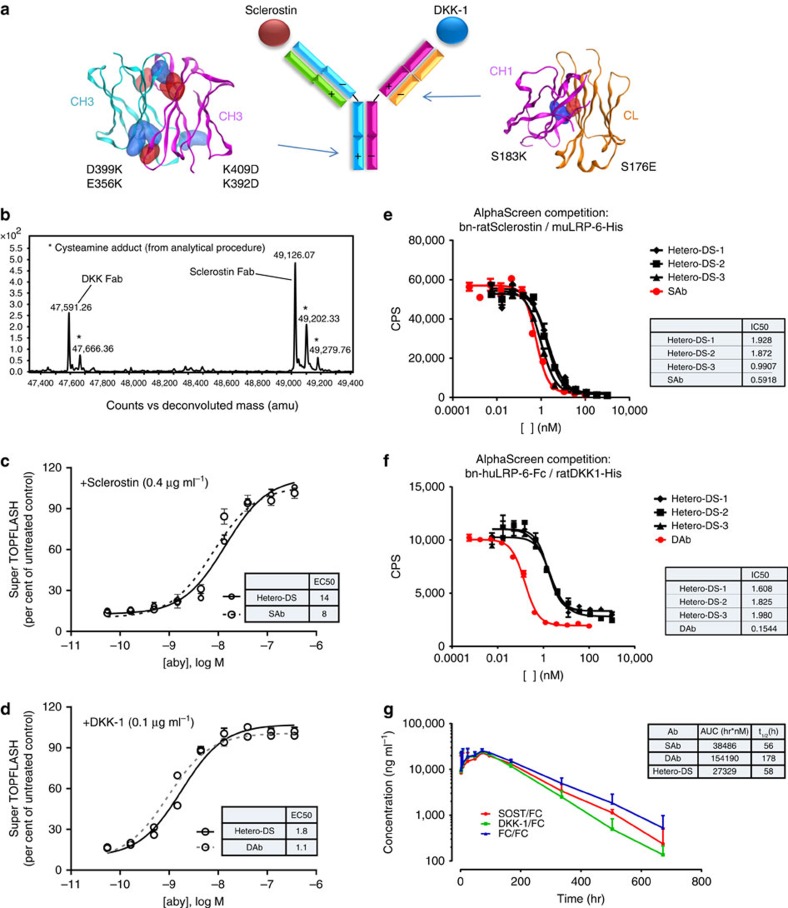
Structure and properties of an engineered bispecific heterodimeric antibody (Hetero-DS) targeting sclerostin and DKK-1. (**a**) Hetero-DS generation utilizing charge pair mutations. Two charge pair mutations as shown are introduced at the CH3 domain interface to drive heavy-chain heterodimerization. In order to promote cognate light- and heavy-chain pairing, a single charge pair mutation at the CL-CH1 interface is introduced (v1, shown here). In another Hetero-DS version (v2), an additional charge pair mutation at the VL-VH interface is introduced. (**b**) Non-reduced mass spectrometry analysis of a Hetero-DS subjected to papain proteolysis. The Fab fragment analysis demonstrates cognate pairing of light chain and heavy chain and no detectable mispaired impurities. The additional peaks near the Scl-Ab and DKK1-Ab Fab mass are due to heterogeneous cleavage of Fab during proteolysis treatment. (**c**) The cell-based bioactivities of the Hetero-DS and the parent sclerostin antibody to human sclerostin (*n*=3). The EC50s are depicted in the insert table. (**d**) The bioactivities of the Hetero-DS and the parental DKK-1 antibody to human DKK-1. Data are from three independent experiments). The EC50s are depicted in the insert table. (**e**,**f**) AlphaScreen competition analysis shows the ability of Hetero-DS to disrupt the interaction of sclerostin with lipoprotein-related protein 6 (LRP6) and disrupt the interaction of DKK-1 with LRP6. Data represent at least three experiments. (**g**) Rat pharmacokinetic profile of Hetero-DS using three intact and total assays.The exposures (AUC) and terminal half-life (*t*_1/2_) of the human Hetero-DS and their corresponding parental sclerostin and DKK1 antibodies are shown in the insert table. Data are from one experiment with 3 rats and are presented as mean+s.e.m.

To generate a human sclerostin and DKK-1 bispecific antibody using this approach, three human Scl-Abs (Ab1, Ab2 and Ab3) were combined with two human DKK-1 antibodies (DAb1 and DAb2) to engineer and produce 10 different bispecific Hetero-DSs on an IgG2 backbone (Fig. 3a and Supplementary Fig. 3). These 10 candidates were transiently expressed, purified and subsequently characterized by conducting binding and bioactivity assays and manufacturability assessments, resulting in three final candidates.

**Figure 4:**
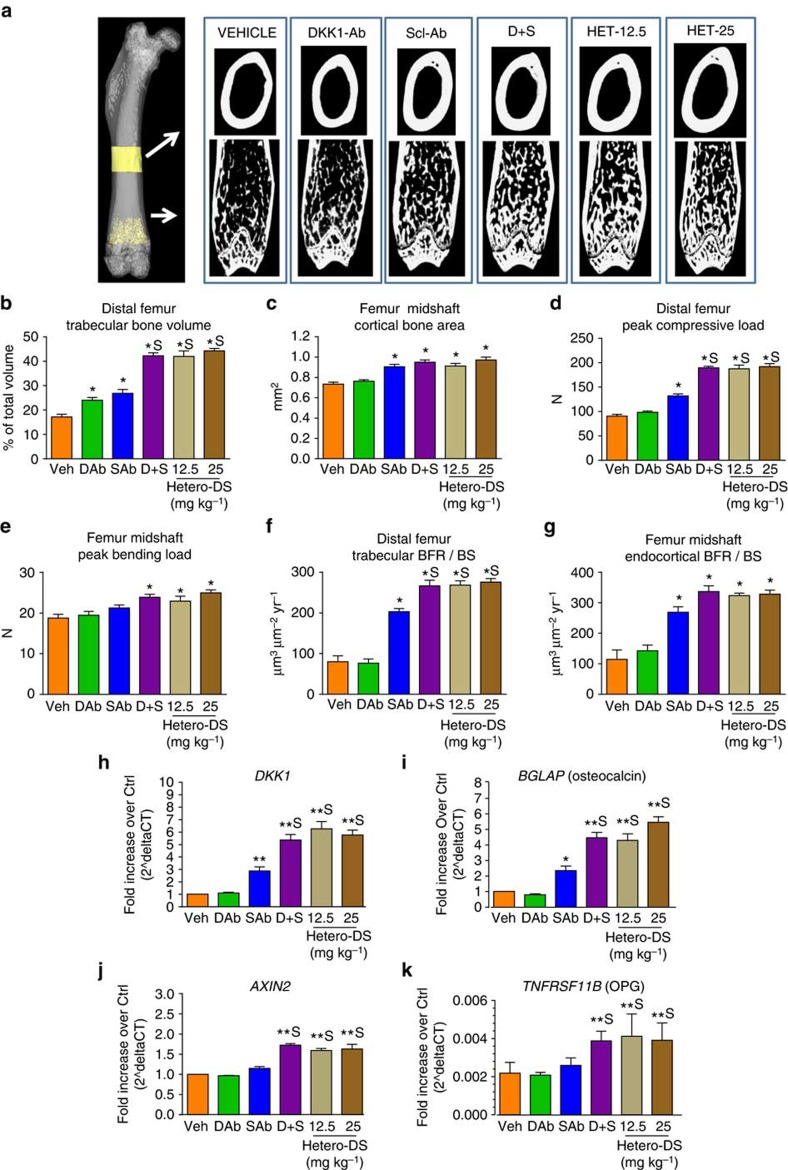
The bispecific heterodimeric antibody Hetero-DS increased bone formation, bone mass, and bone strength in mice. Ten-week-old male B6D2F1 mice were injected subcutaneously twice weekly with vehicle (Veh), sclerostin antibody (Scl-Ab; 12.5 mg kg^−1^), DKK-1 antibody (DKK1-Ab; 12.5 mg kg^−1^), Scl-Ab+DKK1-Ab (S+D; 12.5 mg kg^−1^ each) or Hetero-DS (12.5 and 25 mg kg^−1^) for 3 weeks (*n*=6/group). (**a**) Representative microCT images of the femur midshaft and distal femur metaphysis for each group. (**b**) Trabecular BV in the distal femur and (**c**) cortical area at the midshaft were measured by microCT. Peak load was determined by compression testing and three-point bending at the (**d**) distal femur and (**e**) femur midshaft, respectively. BFR (surface referent) (BFR/BS) was determined by histomorphometry at the (**f**) cancellous distal femur and (**g**) femur midshaft endocortex. Taqman gene expression analysis of Wnt/β-catenin target genes in the lumbar vertebrae of mice treated with Hetero-DS (*n*=6 per group) (**h**–**k**). Genes of interest shown were normalized to a housekeeping gene (*HPRT*). Data represent one experiment with 6 animals per group and are presented as mean±s.e.m. *P<0.05 versus vehicle, ^s^*P*<0.05 versus Scl-Ab; One-way analysis of variance, Tukey's *post hoc* test.

Attributes that were examined for these three candidates after transient production in 293-6E cells included structural, chemical and biological photostabilities and biological properties. The bispecific antibodies demonstrated (1) high expression levels following transient expression (Supplementary Fig. 4a), (2) high purity as measured by size exclusion chromatography (Supplementary Fig. 4b), (3) correct pairing of light and heavy chains as assessed by mass spectrometry (Fig. 3b) and (4) slightly lower thermal and storage stabilities (Supplementary Fig. 4c,d). Thus, the charge pair mutations did not impair important production attributes and the heterodimeric protein retained most of the parent antibody attributes. The human Hetero-DS had potent *in vitro* binding affinities to sclerostin and DKK1 as measured by KinExA (Supplementary Fig. 5b,c). The human Hetero-DS bound human sclerostin and DKK-1 with an apparent *K*_D_ of 42 and 702 pM, respectively. These binding affinities were comparable to the parental antibodies Scl-Ab (36 pM) and DKK1-Ab (281 pM). Since Hetero-DS has one sclerostin heavy chain–light chain pair and one DKK-1 heavy chain–light chain pair, it is expected that the final product will bind monovalently to one sclerostin and one DKK-1 molecule. To address whether binding to different ligands could occur simultaneously, we showed Hetero-DS simultaneously bound both DKK-1 and sclerostin by performing a dual antigen-binding ELISA (Supplementary Fig. 5b). The human Hetero-DS also blocked the interaction of DKK-1 and sclerostin with LRP6, and neutralized DKK-1 and sclerostin resulting in activation of osteoblast canonical Wnt signalling with similar potency to parental antibodies (Fig. 3c–f and Supplementary Fig. 5a,b). These results show that the heterodimeric antibody has potent *in vitro* binding and neutralizing activity for both sclerostin and DKK-1.

In rats, the pharmacokinetic profile of the human Hetero-DS was similar to that of the parental Scl-Ab (Fig. 3g, inset), whereas DKK1-Ab had a greater half-life. These results indicate that the molecule had good *in vivo* stability and showed no signs of biotransformation as shown in Fig. 3.

### Hetero-DS increases bone mass and strength in mice

The bone-forming effects of the bispecific antibody were examined by micro-computed tomography (microCT) analysis in 10-week-old intact mice treated for 3 weeks with human Hetero-DS. The Hetero-DS resulted in robust increases in BV and bone strength in the distal femur metaphysis that were significantly greater than those observed with vehicle or monotherapy treatment. Notably, despite the fact that Hetero-DS has monovalent binding to each target, a 12.5 mg kg^−1^ dose showed equivalent effects to the combination of 12.5 mg kg^−1^ of Scl-Ab and 12.5 mg kg^−1^ DKK1-Ab (Fig. 4a–e). At the femur diaphysis, Scl-Ab, S+D and hetero-DS similarly increased BV and strength compared with vehicle controls. Importantly, bone strength increased proportionately to bone mass at both sites (Supplementary Fig. 6) with no increase in woven bone, suggesting that bone quality was maintained. Consistent with these findings, BFR/BS was greatest in the bispecific and combination groups at these sites (Fig. 4f,g and Supplementary Table 2). At the distal femur, the Hetero-DS-mediated increase in BFR/BS was associated with an increase in the extent of bone formation (mineralizing surface [MS/BS]), while at the femur endocortical surface, mineral apposition rate was increased. Although distal femur ES/BS was not affected by either monotherapy, Hetero-DS resulted in a significant reduction relative to controls (Supplementary Table 2).

To examine the mechanism by which dual inhibition of sclerostin and DKK-1 promoted bone formation in these mice, gene expression was analysed in whole-bone tissue lysate prepared from lumbar vertebrae (L1). Dual inhibition of sclerostin and DKK-1 robustly increased classical Wnt/β-catenin transcriptional targets (*DKK1* and *AXIN-2*) and markers of both osteoblastogenesis (*BGLAP,* osteoprotegerin *[OPG]*, and *RUNX2*) and osteocyte activity (*SOST* and *MEPE*) relative to vehicle and monotherapy controls (Fig. 4h–k and Supplementary Fig. 7a). Furthermore, treatment with Scl-Ab and Hetero-DS resulted in a compensatory increase in other secreted Wnt antagonists examined including WIF1 and SFRP4, suggesting feedback regulation involves multiple negative regulators besides Sclerostin and DKK-1 in bone tissue (Supplementary Fig. 7b). The increases in osteogenic gene expression were consistent with the increased BMD following treatment with Hetero-DS and combination therapy relative to monotherapy (Supplementary Fig. 7c).

### Hetero-DS improves fracture repair in rats

**Figure 5:**
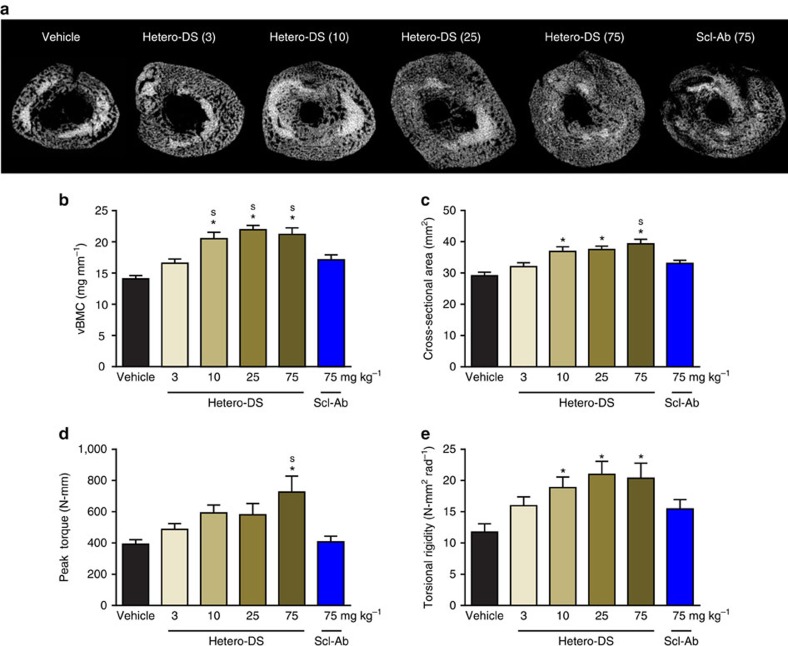
Hetero-DS dose dependently improved fracture repair in a rat closed femur fracture model. Three-month-old male Sprague-Dawley rats underwent closed femur fracture surgery and were injected subcutaneously twice weekly with vehicle, Hetero-DS at 3, 10, 25, or 75 mg kg^−1^ or sclerostin antibody (Scl-Ab) at 75 mg kg^−1^ for 5 weeks (*n*=18/group). (**a**) Representative transverse images of the fractured femur by microCT. MicroCT parameters included (**b**) total volumetric bone mineral content (vBMC) and (**c**) callus cross-sectional area. Healed femurs were tested in torsion to failure and (**d**) peak torque and (**e**) torsional rigidity were measured. Data presented as mean±s.e.m.; *P<0.05 versus vehicle and ^s^*P*<0.05 versus Scl-Ab by ANOVA+Tukey's *post hoc* test.

The enhanced bone-healing effects of dual sclerostin and DKK-1 inhibition were confirmed in a closed femur fracture model in rats using a rat surrogate version of Hetero-DS (rHetero-DS). This molecule exhibited similar *in vitro* biological activity as human hetero-DS with Wnt reporter assay EC50s of 6.15 and 3.04 nM to sclerostin and DKK-1, respectively (Supplementary Fig. 8a). Administration of rHetero-DS for 5 weeks in 3-month-old rats dose dependently increased callus bone volume, cross-sectional area and torsional strength (Fig. 5a–e). A 75 mg kg^−1^ dose of Scl-Ab resulted in lesser, non-significant increases in these parameters similar to those resulting from a 25-fold lower dose of rHetero-DS. Thus, increasing the dose of Scl-Ab could not achieve the bone-healing effect of the rHetero-DS, underscoring its distinct mechanism of action relative to that of DKK-1 inhibition. Similarly, in a five-week rat closed fracture model, DAb had significantly inferior bone-healing effects compared with half a dose of another bispecific candidate (rBsAb2) with similar potency as rat hetero-DS (Supplementary Fig. 8a,b).

### Hetero-DS increases bone formation markers in primates

**Figure 6:**
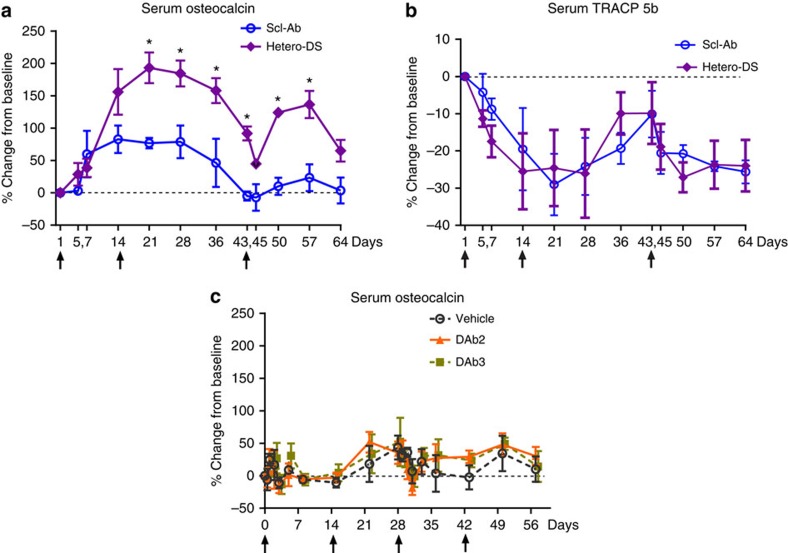
Hetero-DS increased the serum bone formation marker osteocalcin to a greater extent than sclerostin antibody (Scl-Ab) alone in cynomolgus monkeys. Adolescent female cynomolgus monkeys were injected subcutaneously with 25 mg kg^−1^ Hetero-DS or Scl-Ab on days 0 and 14 and by intravenous injection at day 43 (*n*=3 per group) (**a**) Serum osteocalcin measured at time points from 1 to 64 days after the initial injection of human Hetero-DS. Arrows indicate times of injection (first two doses were administered by subcutaneous injection followed by a third intravenous dose). Data presented as mean±s.e.m.; *P<0.05 versus Scl-Ab by two-way analysis of variance with Bonferroni *post hoc* test. (**b**) Serum TRACP5b was measured at time points from 1 to 64 days after the initial injection of human Hetero-DS. (**c**) Serum osteocalcin levels following administration of four subcutaneous doses of human DKK-1 antibodies DKK1-Ab2 (DAB7.5) and DKK1-Ab3 (huDAB10) at 30 mg kg^−1^ each in 9–14-year-old cynomolgus monkeys (*n*=5 per group). Serum osteocalcin was measured at time points from 1 to 56 days after injection. Arrows indicate times of injection. Data presented as mean+s.e.m.; *P<0.05 versus vehicle by two-way analysis of variance+Tukey's *post hoc* test.

To confirm the bone-forming effects of human Hetero-DS in primates, 5-year-old female intact cynomolgus monkeys (cynos) were injected subcutaneously with 25 mg kg^−1^ Hetero-DS or Scl-Ab on days 0 and 14 and by intravenous injection at day 43. Although drug exposure was similar between the Hetero-DS and Scl-Ab groups, the increases from baseline in serum bone osteocalcin, a sensitive and specific marker of bone formation, were significantly greater with Hetero-DS than with Scl-Ab (Fig. 6a). We observed a trend towards decreased TRACP5b, a bone resorption marker that was comparable between Hetero-DS and Scl-Ab (Fig. 6b). In a separate experiment, 9–14-year-old female cynos were administered a 30 mg kg^−1^ dose every two weeks of two human DKK1-Abs with comparable biological activity, DKK1-Ab2 and DKK1-Ab3. Consistent with the lack of a robust bone anabolic activity of DKK-1 antibody in intact mice and rats, we did not observe significant increases in serum osteocalcin in cynos (Fig. 6c) and this could not be attributed to the presence of anti-drug antibodies or lower drug exposure of DKK-1 ab. These results confirmed the robust effect of the heterodimeric antibody on bone formation markers relative to the parental antibodies in primates.

## Discussion

Increased DKK-1 as a result of sclerostin inhibition and known differences in the effects of these Wnt antagonists on various Wnt classes led to investigations of the dual inhibition of DKK-1 and sclerostin on bone formation *in vivo*. Administration of a bispecific heterodimeric antibody targeting sclerostin and DKK-1 resulted in large, rapid increases in bone formation, bone mass and bone strength in intact bones in mice, and fractured bones in rats. Consistent with these findings we showed that Hetero-DS increased Wnt signalling as evidenced by increased expression of classical Wnt/β-catenin transcriptional targets including DKK-1 in bone. These changes were significantly greater than with either Scl-Ab or DKK1-Ab treatment alone. Our rodent data were extended to cynos, where it was confirmed that human Hetero-DS resulted in greater increases in bone formation markers relative to monotherapy.

In the skeleton, there are likely multiple mediators that act to limit bone formation. Both sclerostin and DKK-1 have certain redundant functions by blocking LRP5/6 binding to specific Wnts and downstream Wnt signalling. Inhibition of one of these proteins may engender a compensatory response in the other to return Wnt signalling to a steady state. Indeed, both SOST-deficient mice and Scl-Ab-treated rats^26^ and mice exhibited increases in bone DKK-1, consistent with the reported elevations in serum DKK-1 levels in patients with sclerosteosis^29^. In agreement with this finding, greater expression of canonical Wnt signalling targets was found in the bone of animals treated with Hetero-DS relative to monotherapy. Also, other Wnt antagonists showed compensatory increases in expression suggesting Wnt feedback regulation can involve multiple pathway components. It is not currently known if the compensatory increases in DKK-1 or other factors are related to the attenuation of serum bone formation marker increases observed in rodents^26^ and humans^29^ following chronic Scl-Ab administration. Increased serum DKK-1 was also reported in patients with osteoporosis receiving parathyroid hormone (PTH1–34), coinciding with decreases in serum bone formation markers^30^.

**Figure 7:**
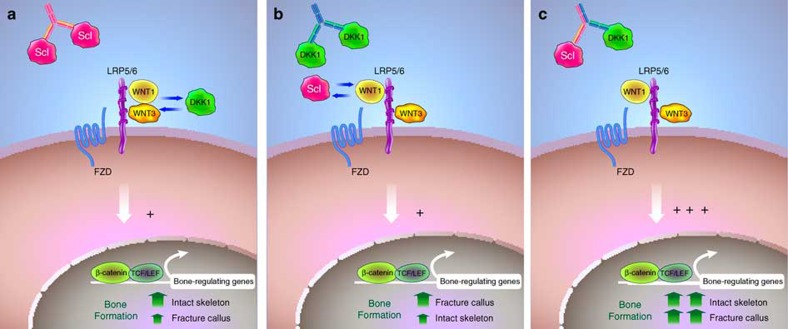
Simplified model for the proposed roles of the Wnt antagonists DKK-1 and sclerostin in the intact and injured skeleton. Canonical WNT-β-catenin signaling is initiated when different classes of Wnt ligands (Wnt1 or Wnt3) bind to distinct β-propeller domains on either LRP5 or LRP6 in complex with Frizzled (FZD). Sclerostin and DKK-1 are secreted Wnt antagonists that inhibit bone formation by directly binding to LRP5/6 receptors and blocking Wnt1 or both Wnt1 and Wnt3 class ligands, respectively. Wnt antagonism by sclerostin and/or DKK-1 is also modulated via interactions with the receptors LRP4 and Kremen, though the functional consequence of these interactions remains to be elucidated. Antibodies that neutralize sclerostin increase bone formation by activating Wnt signalling. Sclerostin plays a dominant role in bone maintenance responding to changes in loading and other cues. In contrast, DKK-1 is normally present at low levels and DKK-1 antibodies have limited bone-forming potential in the intact skeleton (**a**). DKK-1 levels are increased upon injury (and in certain bone diseases) and DKK-1 antibodies improve fracture healing (**b**). In the fracture callus, Scl-Ab increases callus bone density but higher levels of DKK-1 may limit its efficacy (**a**,**b**). Dual inhibition of DKK-1 and sclerostin has synergistic effects in both the intact skeleton and in the fracture repair process by targeting redundant biological activities (**c**). In addition, spatial differences in the expression of DKK-1 and sclerostin in the fracture callus suggest distinct biologic activities of DKK-1 and sclerostin in modulation of the repair process.

The current study is the first to provide comparative data demonstrating the greater bone-forming effects of Scl-Ab versus DKK1-Ab in the intact rodent skeleton, with dual inhibition resulting in further improvements. The bone anabolic activity of Hetero-DS was also superior to Scl-Ab in cynos. We recognize a comparison of Scl-Ab and DKK1-Ab in the two cyno studies has limitations given the different ages of the animals. However, in all studies with young and old rodents as well as mature cynos, DKK1-Ab has not shown robust bone-forming effects. These results are consistent with sclerostin's dominant expression pattern throughout the osteocyte network, while DKK-1 is expressed at lower levels in bone tissue, particularly in mature rodents^23^. DKK-1 expression was elevated early after fracture in cortical osteocytes and at the periosteum, and DKK1-Ab consequently improved both callus size and bridging in the rat closed fracture model. Our studies showed the Hetero-DS dose-dependently improves fracture healing and a low dose had significant effects on callus BV and strength whereas higher doses of Scl-Ab or DKK1-Ab did not show as significant a benefit. Although Scl-Ab alone did improve callus bone mass and strength to some degree, callus size and bridging were not significantly affected, reflecting an augmentation of bone that may be dependent on formed bridges. In contrast, treatment with Hetero-DS resulted in the largest increases in callus bone mass and strength relative to either monotherapy. On the basis of these and other data, the distinct roles of sclerostin and DKK-1 can begin to be delineated. Sclerostin is highly expressed by osteocytes in the adult skeleton and by inhibiting Wnt signalling, it thereby keeps the majority of bone lining cells in a quiescent state as part of normal skeletal maintenance. Sclerostin expression is downregulated when bone formation is required, either after loading^31^ and exposure to hormones such as PTH^32^, or due to osteocytic injury after fracture. Although DKK-1 plays a critical role during skeletal development, it is not highly expressed in adult bone unless activated by an insult^23^ (Fig. 7a–c). The increase in DKK-1 observed in the periphery of the injury site could reflect activation of Wnt signalling and mobilization of periosteal osteochondrogenic progenitor cells previously shown to be required for callus formation^33^.

Bone resorption decreased with Scl-Ab treatment in rodents, primates and humans. The current study demonstrated further decreases in eroded surface, a bone resorption parameter, by histology in both mice and rats after dual inhibition of sclerostin and DKK-1. Whether these results are directly attributable to the greater increases in Wnt signalling or secondary to the high percentage of bone-forming surface remains to be determined. Consistent with the former possibility, we observed increased OPG expression, a transcriptional target of Wnt/β-catenin, following treatment with Hetero-DS or combination therapy. Activation of Wnt signalling could also have a direct effect on osteoclast bone cell lineages^34^. Future studies are needed to address this possibility and further define the complex crosstalk of Wnt pathway components with different types of bone cells and their precursors. The activation of formation and decrease in bone resorption with Hetero-DS is consistent with the effects of Scl-Ab alone, and likely reflect the activation of modelling-based bone formation, which has been reported in rats and cynos with Scl-Ab^35^.

At present, the relative contribution of sclerostin and DKK-1 in injured and disease states remains to be elucidated. Sclerostin acts as a rheostat to inhibit or increase bone mass accrual during normal growth and adulthood. In contrast, DKK-1 is required for development, and elevated levels during disease and injury may play a pathogenic role and/or reflect perturbed Wnt signalling and bone metabolism^23^,^36^. Indeed, previous studies from our group and others' show that increasing *DKK1* gene dosage resulted in a proportional loss of bone accrual and inhibition of bone repair^23^,^37^,^38^. In addition to the partial redundancy of these factors, we observed spatial differences in DKK-1 and SOST expression in the fracture callus that may drive distinct and additive bone inhibitory effects.

We engineered a subcutaneously injectable bispecific antibody with desirable physicochemical properties and high affinity to DKK-1 and sclerostin. Engineering charged pair mutations in the CH1 and Fc regions resulted in a high percentage of the desired bispecific heterodimer and a negligible presence of mispaired species. Furthermore, these mutations did not compromise the thermal stability and cell titres of the protein produced nor did they lead to aggregation, unlike other bispecific antibody formats. Hetero-DS showed similar pharmacokinetic attributes to the parental Scl-Ab, perhaps due to similar target-mediated drug disposition. We demonstrate herein that the Hetero-DS has the potential to address compensatory increases in DKK-1 leading to increased therapeutic efficacy. We anticipate a therapy for fracture repair would involve a limited treatment regimen. In all our studies, administration of Hetero-DS for up to 9 weeks was well tolerated and no adverse clinical signs were observed in rats or cynos. As with any therapeutic candidate, the safety profile of Hetero-DS will be assessed in formal toxicology studies.

In summary, inhibition of DKK-1 and sclerostin targeted unique facets of the repair process including cortical bridging and bone formation of the formed bridges, thereby improving healing to a degree that exceeded that of monotherapy. Future studies would aim to identify tissue-resident and/or circulating cells and progenitors that promote repair and elicit these distinct responses. The studies conducted to date support the therapeutic promise of Hetero-DS for patients with bone disorders and those who suffer frequent or severe fractures.

## Methods

### Animals

All animals procedures described below were conducted in an Association for Assessment and Accreditation of Laboratory Animal Care accredited facility in accordance with the requirements and guidelines of the US National Research Council and complied with the protocols approved by the CRP Institutional Animal Care and Use Committee of the institutions described below. These studies were performed at Amgen unless described otherwise below, with *ex vivo* analysis generated by Amgen. Sample sizes for the rodent studies below were driven by knowledge gained from previous published studies with Scl-Ab, with larger n required for fracture healing studies that exhibit high variability (*n*=14–18/group) relative to bone pharmacology studies with end points that are less variable (*n*=6–10/group). The n of the cynomolgus monkey studies were limited to four to five per group based on the expected consistency of serum biomarker responses and a desire to minimize the number of monkeys required.

### DKK1 quantitation in sclerostin-deficient animals

Tibiae were collected from wild-type and SOST knockout mice (4.5- and 6.5-months old) and OVX rats treated with Scl-Ab for assessment of DKK1 mRNA and protein. Briefly, 6-month-old Sprague-Dawley rats were OVX and 5 months later were injected subcutaneously twice weekly with Vehicle (A5SuT) or Scl-Ab at 10 or 25 mg kg^−1^ for 5 weeks, with a sham-operated group administered Vehicle (*n*=10/group). Tibia were isolated, cleaned of connective tissues, flash-frozen in liquid nitrogen, and pulverized in frozen stainless steel Bessman Tissue Pulverizers (Catalog #08-418-2, Fisher Scientific, Pittsburg PA) and pulverized according to manufacturer instructions. The resulting tissue powder was transferred to pre-frozen 14 ml polypropylene tubes (BD Falcon, Catalog #352059, Franklin Lakes, NJ) using a pre-frozen scalpel blade For mRNA expression, bone homogenates were prepared according to the Quantigene Sample Processing Kit protocol (Affymetrix, Santa Clara, CA), and extracts were tested for RNA presence and quality by using a Bioanalyzer 2100 (Agilent Technologies, Santa Clara, CA). The mRNA expression level of DKK1 was measured relative to the housekeeping gene HPRT1 using Quantigene Plex 2.0 kits (Affymetrix; Santa Clara, CA, USA) in accordance with manufacturer protocols. Tibial protein content was extracted using a 50 mM Tris buffer, pH 7.4, containing 0.1 M NaCl and 0.1% Triton X-100. Protein concentration in individual extracts was evaluated using a standard BCA Protein Assay (Pierce Co., Rockford, IL). DKK1 concentration in rodent protein extracts was evaluated using a human/mouse/rat Luminex-based single-plex kit (Millipore, Billerica, MA) according to the manufacturer's protocol and normalized to the total protein concentration.

### Bone pharmacology studies

In the first study, 6-month-old Sprague-Dawley rats underwent ovariectomy and 2 months later were injected subcutaneously twice weekly with Vehicle (T8SuT), Scl-Ab (18.2 mg kg^−1^), DKK1-Ab (18.2 mg kg^−1^) or Scl-Ab+DKK1-Ab (S+D; 18.2 mg kg^−1^ each) for 5 weeks, with a sham-operated group-administered vehicle (*n*=10/group). DXA was performed on the left leg and at the lumbar spine (L1–L5) at treatment baseline and before termination, with areal BMD expressed as a percentage change from baseline (QDR 4500a, Hologic, Bedford, MA). Undecalcified parasagittal 4-μm-thick sections of LV2 and 6-μm-thick transverse sections of the left tibia at the tibiofibular junction were prepared, and histomorphometry was performed as previously described^39^. Measurement of BFR/BS was based on fluorochrome labels (20 mg kg^−1^ calcein subcutaneously) administered 3 and 13 days before termination. BFR/BS was calculated as the product of MS/BS and mineral apposition rate. Other bone endpoints quantified by histomorphometry included BV/TV, Tb.Th, Tb.N and ES/BS at the vertebra (trichrome-stained slides), and cortical area and thickness at the tibia diaphysis.

In the second study, 10-week-old male B6D2F1 mice were injected subcutaneously twice weekly with vehicle, Scl-Ab (12.5 mg kg^−1^), DKK1-Ab (12.5 mg kg^−1^), hetero-DS (12.5 and 25 mg kg^−1^), or Scl-Ab+DKK1-Ab (S+D; 12.5 mg kg^−1^ each) for 3 weeks (*n*=6/group). At termination, right femurs were collected for microCT analysis and strength testing in three-point bending and left femurs were collected for histomorphometry. Whole femora were scanned (80 kV and 80 μA; GE Locus SP, GE Healthcare, Pittsburgh, PA) and reconstructed at 8 μm isotopic resolution. Regions of interest (10% of femur height) were selected for analysis in the distal cancellous metaphysis (thresholds ranged from 468 to 706 mg cm^−3^) and the central cortical diaphysis (threshold: 920 mg cm^−3^) to quantify trabecular BV/TV and cortical area, respectively (MicroView v.2.2). Destructive mechanical testing was performed at the femur midshaft in three-point bending (6.0 mm span length), after which planoparallel segments (2.0 mm in height) were trimmed using a diamond wire saw and axially compressed to failure. Both tests were performed on a servohydraulic test system (MTS Mini-Bionix II) at a rate of 6.0 mm min^−1^ with peak load recorded. Histomorphometry was performed at the distal femur metaphysis and femur midshaft as described above.

In a third study performed at Covance Research Products (Alice, TX, USA), adolescent 4–6-year-old female cynos (*Macaca fascicularis*) were socially housed in wall-mounted cages (two to three animals per cage) equipped with an automatic watering system. All animals had access to 2050C Certified Global Primate Diet (PMI Nutrition International, Inc., Shoreview, MN) twice daily, containing 0.93% calcium, 0.75% phosphorus and 8.0 IU of vitamin D per gram, as well as daily food supplements including fresh fruit. The animal room environment was controlled, with settings targeted at temperature 24±3 °C, humidity 50±20%, 12-hour light/dark cycles, and 12 air changes per hour. An acclimation period of 6 weeks was allowed before the start of test article administration. Only animals considered in good health and with normal serum/urine chemistry panels were used in the study. Animals were randomized to treatment groups based on body weight and the serum bone formation marker osteocalcin. Vehicle (PBS, *n*=2), Scl-Ab (*n*=4) or human Hetero-DS (*n*=5) at 25 mg kg^−1^ was administered subcutaneously on days 1 and 14 and intravenously on day 43 in a volume of 1 ml kg^−1^. Serum samples were collected approximately once weekly through day 64; ELISAs were performed to determine drug concentrations and the bone formation marker osteocalcin (N-MID Osteocalcin; Nordic Bioscience, Herlev, Denmark) and TRACP 5b (IDS, Scottsdale, AZ) were measured at each time point. In a separate study in more mature (9–14-year old) cynos performed at Charles River Labs (Montreal, Canada), vehicle or 30 mg kg^−1^ of either DKK1-Ab2 (DAb7.5) or DKK1-Ab3 (DAb10) was administered by SC injection every 2 weeks for 8 weeks, with frequent serum sampling to day 57 (*n*=5/group). At each time point, drug concentrations and the bone formation marker osteocalcin were measured by ELISA.

### Fracture repair models

In the first study, 7-month-old male Sprague-Dawley rats underwent closed femur fracture surgery^24^ and were injected subcutaneously twice weekly with vehicle, Scl-Ab (25 mg kg^−1^), DKK1-Ab (25 mg kg^−1^) or Scl-Ab+DKK1-Ab (S+D; 25 mg kg^−1^ each) for 7 weeks (*n*=14–18/group). At termination, the intramedullary pins were removed and the fractured femurs were scanned and analysed by microCT as previously described^24^. Briefly, the central 1 mm of the fracture callus was delineated from the pre-existing cortex and the BV/TV in this region was measured after thresholding. In addition, the fracture bridging was assessed at the fracture line on two sets of orthogonal longitudinal image pairs, which captured bridging every 45° around the circumference of the bone. For each image, two observers scored the presence of a bone bridge on each of four points spanning both the inner cortex and external callus, with the results averaged and expressed as a percentage to total bridging for each sample. Repair strength was assessed in three-point bending as described for the intact rat femur above.

In the second study conducted at PharmaLegacy Labs (Shanghai,China), 10-week-old male Sprague-Dawley rats underwent closed femur fracture surgery and were injected subcutaneously twice weekly with vehicle, rat Hetero-DS at 3, 10, 25, or 75 mg kg^−1^, or Scl-Ab at 75 mg kg^−1^ for 5 weeks (*n*=18/group). MicroCT was performed at the fractured femur after pin removal as described above. Repair strength was determined in torsion to failure at 2° per second after both ends of the femur were embedded in square pots using dental cement to generate test samples spanning ∼12 mm in height. Torsional endpoints included peak torque and torsional rigidity (torsional stiffness × span height/180*π*).

In the third study conducted at PharmaLegacy Labs (Shanghai, China), 3-month-old rats underwent closed femur fracture surgery and were injected subcutaneously twice weekly with Bispecific Ab #2 (rBspAb2), DAb and SAb at 0.172 μmol kg^−1^, 0.33 μmol kg^−1^ and 0.33 μmol kg^−1^ respectively for 5 weeks (*n*=18/group). DXA was performed at the central 30% of fractured femur after pin removal (PIXImus II; GE Lunar, Madison, WI, USA).

### Serum bone formation marker analysis

Blood samples were collected pre-dose (day 1) and during the treatment phase (days 5, 7, 14, 21, 28, 36, 43, 45, 50, 57 and 64) as well as for two control animals on days 3, 14, 28, 57 and 64. The bone formation marker osteocalcin was measured in all serum samples using a Microvue Osteocalcin ELISA kit (Quidel, San Diego, CA) according to the manufacturer's protocol. The effect of test articles on serum osteocalcin concentration was evaluated by comparison with pre-dose (day 1), and results were presented as per cent change from baseline. Statistical analysis of the per cent change from baseline was performed by using two-way analysis of variance followed by Bonferroni *t*-test for group mean comparisons between the Scl-Ab- and human Hetero-DS-treated groups. All comparisons were conducted using a two-sided test at the 5% significance level. Significant results are reported as *P*≤0.05, where *P* represents the observed probability. All the statistical analyses were performed using GraphPad Prism software.

### Hetero-DS engineering

To identify domain–domain interface residues between light and heavy (Fab region) and heavy-heavy (Fc region) chain, Protein Data Bank was examined to identify high resolution (2.0 Å or better) Fc and Fab crystal structures. Two methods were used to identify the residues involved in the VH/VL, CH1/CL, and CH3/CH3 domain interactions: (1) contact as determined by distance limit criterion and (2) solvent accessible surface area (ASA) analysis. According to the contact-based method, interface residues are defined as residues whose side-chain heavy atoms are positioned closer than a specified limit (4.5 Å) from the heavy atoms of any residues in the second chain. The second method involves calculating solvent ASA of the residues in the presence and absence of the second chain^40^. The residues that show difference (>1 Å^2^) in ASA between the two calculations are identified as interface residues. Both methods identified a similar set of interface residues. These residues were further examined for structural conservation. For this purpose, multiple known Fab and Fc crystal structures identified from the Protein Data Bank were superimposed and analysed by calculating the r.m.s.d. for the side-chain heavy atoms. In the next step, the identified structurally conserved residues were mutated to either negatively charged residues (Asp or Glu) or positively charged residue (Lys) in such a way that the electrostatic interaction drove desired heterodimer formation (that is, heavy-heavy heterodimer and cognate heavy–light-chain pairing) and discouraged undesired homodimer products^41^,^42^. For example, in order to drive specific pairing of light and heavy chain, negatively and positively charged residue mutations were introduced in the DKK1 light and heavy chains, respectively, and vice versa for Sclerostin light and heavy chains. Structurally conserved and buried residue positions were given preference for the mutations. The domain interface-buried (% ASA≤10; % ASA refers to ratio of observed ASA to the standard ASA of amino acid) and exposed (% ASA>10) residue positions were identified using the solvent ASA calculation.

Bispecific hetero-DSs were constructed from the polypeptide chains of existing sclerostin and DKK1 antibodies that are engineered with CPMs to select for the correctly assembled heterodimerized form. The sclerostin humanized antibodies Ab1, Ab2 and Ab3 and rat Scl-Ab were combined with fully human DKK1 antibodies DAB7.4 and DAB7.5 and DAB10 to engineer and produce 10 different human Hetero-DSs and a rat hetero-DS. Human Hetero-DSs were engineered in an IgG2 backbone due to its lower propensity to trigger antibody-dependent cellular cytotoxicity compared with IgG1. Different antibody combinations were tested with either v1 (heavy chain 1: S183K, K392D, K409D; heavy chain 2: S183E, E356K, D399K; light chain 1: S176E; light chain 2: S176K) or v2 (heavy chain 1: Q39K, S183K, K392D, K409D; heavy chain 2: Q39E, S183E, E356K, D399K; light chain 1: Q38E, S176E; light chain 2: Q38K, S176K) CPMs and the corresponding light-chain subtypes. By using a variety of antibody, CPM, and light-chain subtype combinations, an empirical approach was taken to determine which combination had the desired potency, correct assembly, and manufacturability. The 10 hetero-DS constructs were expressed, purified, and subsequently characterized by conducting binding/bioactivity assays and manufacturability assessments to identify a final candidate for pharmacology studies.

### Protein production and analysis

Antibody constructs were transfected into 2936E (National Research Council of Canada). Briefly, 5–20 L of 293-6E cells were grown to 1.5e6 cells mL^−1^ in F17 media (Life Technologies, Grand Island, NY) in 5 l Optimum growth flasks (Thompson Instrument Company, Oceanside, CA) or a WAVE Bioreactor (GE) and transfected with 0.5 mg ml^−1^ of total plasmid DNA, or 0.125 μg ml^−1^ of each chain^43^. Cells were incubated for 6–7 days at 36 °C. Media was clarified by centrifugation and filtration over a 0.2 μM filter (Corning, Corning, NY). Hetero-DSs were purified using affinity chromatography on Protein A, followed by hydrophobic interaction chromatography using Phenyl High Performance Resin (GE Healthcare, Pittsburgh, PA), followed by cation exchange using Sulphopropyl High-Performance Resin (GE Healthcare, Pittsburgh, PA). The antibodies were dialyzed and concentrated in formulation buffer (10 mM Sodium Acetate, 9% Sucrose, pH 5.2 with 0.004% Tween 20). To assess the Hetero-DS bispecific antibody formation, the material was subjected to mass spectrometry analysis. Non-reducing mass analysis confirmed that the antibody product had two different light chains and two different heavy chains. To confirm the specific light-heavy chain pairing, the Fab fragments were generated through proteolysis (Fab Micro Preparation Kit, Pierce, Grand Island, NY). The mass analysis showed only two species, one corresponding to the DKK1 antibody heavy- and light-chain pairing and the other corresponding to that of a Scl-Ab. The mass analysis confirmed the presence of heterodimeric antibodies having the correct pairing of light and heavy chains in both arms of the sclerostin/DKK1 heterodimeric antibody. The mass analysis indicated the presence of a single species of antibody in the purified sample, with the observed mass matching the calculated mass of the heterodimeric antibody. The resulting heterodimeric antibody had two light chains and two heavy chains, with the heavy chain of the anti-sclerostin portion of the heterodimeric antibody having an S183E (EU) substitution in the CH1 domain and the light chain of the anti-sclerostin portion having an S176K (EU) substitution in the CL domain. The heavy chain of the anti-DKK1 portion of the heterodimeric antibody had an S183K (EU) substitution in the CH1 domain and the light chain of the DKK1 portion had an S176E (EU) substitution in the CL domain.

### Characterization of binding activity

A KinExA 3200 instrument (Sapidyne Instruments Inc., Boise, ID) was used to measure dissociation equilibrium constants for the Hetero-DS/sclerostin/DKK1 interactions. For all experiments, hetero-DS-coated polymethyl methacrylate beads were used as a capture reagent. Adsorption coating of 200 mg of beads was performed using 45 μg of protein at room temperature for 1 h. The protein solution was then removed, and beads were blocked using PBS with 1% bovine serum albumin (BSA) for 2 h at room temperature. Beads were stored in blocking buffer at 4 °C and used within two weeks. All binding reactions were carried out in PBS with 1 mg ml^−1^ BSA, heparin and 0.02% sodium azide at room temperature. For each equilibrium binding study, DKK1 or sclerostin at three fixed concentrations was incubated with titrations of the testing molecules (Ab or hetero-DS) at room temperature for at least 8 h before being flowed over Hetero-DS-coated beads. Free DKK1 or sclerostin in the equilibrated samples was captured on the hetero-DS-coated beads and then detected using a 500 ng ml^−1^ solution of anti-His_6_ Dylight 649 antibody (Rockland Immunochemicals Inc., Limerick, PA). Each sample was measured twice, and data from the three independent equilibrium binding experiments were globally analysed using n-curve analysis in the KinExA Pro 3.6.2 software (Sapidyne Instruments Inc., Boise, ID) to obtain the *K*_d_ value.

### Dual antigen-binding ELISA assay

The 96-well plates were coated with 20 ml per well of 1 mg ml^−1^ of mouse anti-huScl MAb 56H2 in coating buffer (0.015 M Na_2_CO_3_, 0.035 M NaHCO_3_, pH 9.6) and incubated at room temperature for 1 h or 4 °C overnight. Plates were washed once with 100 ml/well of PBST (PBS containing 0.2% Tween20) and then blocked with PBS containing 1% BSA, 1% goat serum and 0.5% Tween20 at 100 ml per well for 1 h at room temperature with shaking. After blocking, hu-Scl (10 ng ml^−1^ diluted in blocking solution) was added at 20 ml per well and incubated at room temperature for 1 h with shaking. Plates were washed once with 100 ml per well of PBST, followed by adding 20 μl per well of diluted bispecific antibodies (0, 0.008, 0.04, 0.2, 1, 5, 25, 125 and 625 nM), parental antibodies, and control IgG in blocking solution and incubated at room temperature for 1 h with shaking. After washing with PBST, 20 μl per well of huDKK1-biotin (10 ng ml^−1^) diluted in blocking solution was added followed by incubation at room temperature for 1 h. After washing with PBST, 20 μl per well of 1:50 000 Neutravidin-HRP (Pierce, Grand Island, NY) diluted in blocking solution was added and incubated at room temperature for 1 h. The plates were washed three times with 100 μl per well of PBST, and 20 μl per well of a SuperSignal ELISA Femto (Thermo, Grand Island, NY) working solution was added and incubated for 2 min. The chemiluminescent signal was measured using an LmaxII Luminometer (Molecular Devices, Sunnyvale, CA) at 425 nm.

### Osteoblast Wnt reporter assays

The ability of the engineered bispecific antibodies to neutralize both DKK1 and sclerostin inhibition of Wnt signalling was assessed using a Wnt1-induced T-cell factor /LEF luciferase reporter assay in osteoblasts. The osteoblast MC3T3E1/TetON-Wnt1/STF-Luc cell line was engineered by lentiviral transduction with a T-cell factor-responsive luciferase construct, a Tet repressor construct, and a doxycycline-inducible Wnt1 construct. In this assay, addition of doxycycline (10 ng ml^−1^) to the culture medium for 24 h induced expression of Wnt1, resulting in the expression of the luciferase reporter gene. MC3T3E1/TetON Wnt1/STF-Luc cells were incubated in the presence of sclerostin and/or DKK1, and Wnt signalling was inhibited due to competitive binding of sclerostin and DKK1 to LRP5/6. Human DKK1 protein (0.1 μg ml^−1^) or human sclerostin protein (0.4 μg ml^−1^) were premixed with control PBS or a serial dilution of the bispecific antibodies. The luciferase signal was determined on the EnVision apparatus (PerkinElmer, Waltham, MA) after 24 h.

### LRP6/DKK1 and LRP6/sclerostin AlphaScreen inhibition assay

An AlphaScreen assay (PerkinElmer, Waltham, MA) was established to measure the effect of human Hetero-DS on either sclerostin or DKK1 binding to LRP6. Dose–response curves were generated by first serially diluting Hetero-DS (1:3 dilutions) in assay buffer (40 mM sodium HEPES pH 7.5, 100 mM NaCl, 1 mM CaCl_2_, 0.1% BSA, 0.05% Tween20), last point containing buffer only. Next, 2 μl of each hetero-DS dilution was transferred from the dilution plate into a 384-well, reduced volume, white, Greiner microtiter assay plate. Next, 2 μl of biotin-labelled human or rat recombinant sclerostin (2 nM) or DKK1 (0.5 nM) was added to the microtiter plate, followed by the addition of 2 μl of recombinant huLRP-6/Fc (3 nM for sclerostin or 0.3 nM for DKK1) and AlphaScreen ‘donor' streptavidin and ‘acceptor' protein A beads (10 μg ml^−1^ each) (PerkinElmer, Waltham, MA). All dilutions were made in the above buffer. The microtiter plate was then sealed and incubated overnight at 20 °C. Inhibition was measured as a decrease in chemiluminescent signal as measured on the EnVision (PerkinElmer, Waltham, MA), using excitation at 680 nm and emission at 520–620 nm.

### Differential scanning calorimetry analysis

Differential scanning calorimetry measurements were obtained using a VP-Capillary differential scanning calorimetry system (Microcal Inc., Northampton, MA) in which temperature differences between the reference cell and the sample cell are continuously measured and calibrated to power units. This data channel is referred to as the DP signal, or the differential power between the reference cell and the sample cell. The unfolding of a protein molecule appears as an endothermic transition or multiple transitions and can be characterized by the thermal transition midpoints (*T*_m_*1*). The protein samples were diluted to 1.0 mg ml^−1^ using the corresponding buffer, while the same buffer was used in the reference cell. The samples were scanned from 10 to 100 °C at a rate of 60 °C per hour with an initial 15 min of equilibration at 10 °C. A filtering period of 10 s was used and the data were analysed using Origin 7.0 software (OriginLab Corp., Northampton, MA). The apparent thermal transition midpoints were reported and the enthalpy of unfolding was obtained using the Origin 7.0 software by integration of the area under the melting curves (AUC).

### Pharmacokinetic studies in rats

Each test article was injected either intravenously or subcutaneously to three Sprague-Dawley rats at 5.0 mg kg^−1^ dosing. Blood samples were taken from each rat, covering times from pre-dose to 672 h post dose. Blood was processed to serum and analysed with conventional pharmacokinetic ELISAs to determine both the total (that retain the Fc scaffold) and intact (that retain sclerostin and DKK1 target binding) concentrations. To measure intact Hetero-DS, two sclerostin and DKK1 intact assays were established. Briefly, half area plates were coated with 1 μg ml^−1^ of human sclerostin (or hu DKK1 for intact assay) in 1 × PBS and incubated overnight at 4 °C, before blocking for 1 h in I-Block buffer. Standards (Stds) and quality control samples (QCs) were prepared in rat serum. Stds, QCs, and serum samples were diluted 1:30 in buffer (1 × PBS, 1 M NaCl, 0.5% Tween20 and 10 mg ml^−1^ BSA), loaded in an ELISA plate, and incubated for 90 min. After a wash step, a horseradish peroxidase (HRP)-conjugated murine anti-human Fc-specific monoclonal antibody (clone 1.35.1, Amgen Inc., Thousand Oaks, CA) was added to each well. After a final wash step, a tetramethylbenzidine peroxidase substrate solution (KPL Inc., Gaithersburg, MD) was added to the wells. The colour development was stopped by addition of 1 M sulfuric acid and the intensity of the colour (optical density, OD) was measured at 450–650 nm. To measure total IgG, a total Fc/Fc bridging ELISA was established. Briefly, half area plates were coated with 2 μg ml^−1^ of anti-human Fc antibody, Mab 1.35.1 in 1 × PBS and incubated overnight at 4 °C. The plates were blocked for at least 1 h by I-Block buffer. Stds and QCs were prepared in rat serum samples under similar conditions as above. Then the ELISA plate was washed and 50 ng ml^−1^ of HRP-conjugated anti-human Fc antibody, Mab 1.35.1, was added, incubated and washed, and the reaction was stopped as described above. Pharmacokinetic profiles were analysed and typical parameters such as half-life and AUC from 0 to infinity (AUC_0-inf_) were estimated.

### RNA extraction and gene expression analysis

RNA was extracted from mouse and rat lumbar vertebrae and femurs from vehicle and treatment groups. Individual tissues were homogenized using TissueLyzer (Qiagen, Valencia, CA) and total RNA extracted and purified by the *mir*Vana total RNA extraction kit (Ambion, Austin, TX). RNA quality and quantity was determined on a NanoDrop spectrophotometer (NanoDrop, Wilmington, DE) and by Bioanalyzer RNA profiling (Agilent Technologies, Santa Clara, CA). RNA was DNAse-treated with DNA-*free* kit (Ambion, Austin, TX) and reverse transcribed according to manufacturer's specifications using random hexamers in the High Capacity cDNA Reverse Transcription Kit (Applied Biosystems, Foster City, CA). Quantitative real-time polymerase chain reaction (qRT-PCR) was performed on cDNA using primers to the housekeeping gene human *ACTB* and the gene of interest's specific exon sequences. Ten-microlitre qRT-PCR reaction components included 1.0 ng μl^−1^ cDNA, 2x Universal PCR Master Mix (Applied Biosystems, Foster City, CA), and gene expression assay (*ACTB*: 75 nM primers, 150 nM probe; EPOR: 300 nM primers, 250 nM probe). The qRT-PCR amplification program was: (1) activation at 50 °C for 2 min; (2) denaturation at 95 °C for 10 min; (3) amplification 40 cycles at 95 °C for 15 s and at 60 °C for 1 min with fluorescence capture at each step (ABI PRISM 7900HT Sequence Detection Systems, Applied Biosystems, Foster City, CA). Threshold cycle values (*C*_T_) were determined using Sequence Detector software version 2.3 (Applied Biosystems, Foster City, CA) and transformed to 2^−ΔCT^ for relative expression of EPOR-specific transcript to ACTB.

Probes were labelled with FAM (5′), VIC (5′) and TAMRA (3′). Primers for murine *RUNX2, DKK1, SOST, OPG, MEPE, AXIN2* and *ALPL* were obtained from Applied Biosystems. A custom primer for murine *BGLAP* (osteocalcin) was designed with the following sequence: 5′-TGAGGACCATCTTTCTGCTCACT-3′ (forward) and 5′-GGCATCTGTGAGGTCAGAGAGA-3′ (reverse).

### *In situ* hybridization and immunohistochemistry

Isotopic ISH was performed using ^33^P riboprobes transcribed from cDNA templates for rat SOST and DKK1. cDNA templates containing portions of the coding sequence of SOST (Genbank #AF326741; nucleotides 32–252) or DKK1 (Genbank #NM_001106350; nucleotides 583–928) were generated by PCR. The DKK1 PCR template incorporated flanking RNA polymerase promoters, while the SOST construct was cloned into the pPCR-ScriptA vector (Agilent Technologies, Santa Clara, CA) that contained flanking RNA polymerase promoters.

Five-micron-thick sections from formalin-fixed, decalcified and paraffin-embedded rat femurs were deparaffinized and hydrated through graded ethanols. Sections were then subjected to deproteination (0.2 M HCl), proteinase K treatment (1 μg ml^−1^), acetylation (0.25% acetic anhydride in 0.1 M triethanolamine), dehydration through graded ethanols and pre-hybridization with hybridization buffer for 2 h in a humidified chamber at 56 °C. After pre-hybridization, 1.5 × 10^6^ c.p.m. of ^33^P-labelled probe (50 μl per slide) was applied to each section and the slides incubated in a humidified chamber at 56 °C for 16 h. Subsequently, sections were washed in successive stages in 4 × and 2 × SSC (3 M NaCl, 300 mM trisodium citrate pH 7.0 at 55 °C), treated with 20 μg ml^−1^ RNaseA at 37 °C, and washed in decreasing SSC concentrations at room temperature until the final stringency of 0.1x SSC at 55 °C. Sections were then dehydrated through graded ethanols containing 300 mM ammonium acetate, air dried and coated with Kodak NTB emulsion, and stored in light tight boxes at 4 °C for 3 weeks. The slides were developed in Kodak D19 developer, fixed in Kodak Fixer, and counterstained with hematoxylin and eosin.

IHC was performed on 5 μm formalin-fixed paraffin-embedded sections. Deparaffinized tissue sections were heat-treated for ∼16–18 h at 60 °C with Antigen Retrieval Citra Solution (Biogenex, San Ramon, CA). Sections were blocked with CAS BLOCK (Zymed Laboratories, Grand Island, NY) and then incubated with goat anti-mouse DKK-1 (H-120) antibody (Santa Cruz Biotechnology, Santa Cruz, CA). For sclerostin IHC, deparaffinized tissue sections were blocked with 5% donkey serum and incubated with goat anti-mouse Scl-Ab (R&D Systems, Minneapolis, MN), followed by incubation with a biotin-conjugated donkey anti-goat IgG (Jackson Labs, Sacramento, CA). Tissue sections were quenched with 3% peroxidase solution and detected with Envision Labelled Polymer HRP Mouse (DAKO Corp, Carpinteria, CA; cat. # K4001) or with Vectastain Elite ABC kit (Vector Labs, Burlingame, CA). Sections were immersed in high pH (9.5) Tris buffer for up to 5 min to reduce nonspecific chromogen binding before reaction sites were visualized with DAB+ Substrate-Chromagen System (DAKO Corp., Carpinteria, CA) and counterstained with hematoxylin.

## Additional information

**How to cite this article:** Florio, M. *et al*. A bispecific antibody targeting sclerostin and DKK1 promotes bone mass accrual and fracture repair. *Nat. Commun.* 7:11505 doi: 10.1038/ncomms11505 (2016).

## Supplementary Material

Supplementary Information Supplementary Figures 1-8 and Supplementary Tables 1-2
