# Supplementary material for: A bispecific antibody targeting sclerostin and DKK-1 promotes bone mass accrual and fracture repair
Source: Nat Commun. 2016 May 27;7:11505. doi: 10.1038/ncomms11505 (PMC4894982; doi:10.1038/ncomms11505)
Supplement: Supplementary Information — Supplementary Figures 1-8 and Supplementary Tables 1-2 [file ncomms11505-s1.pdf]

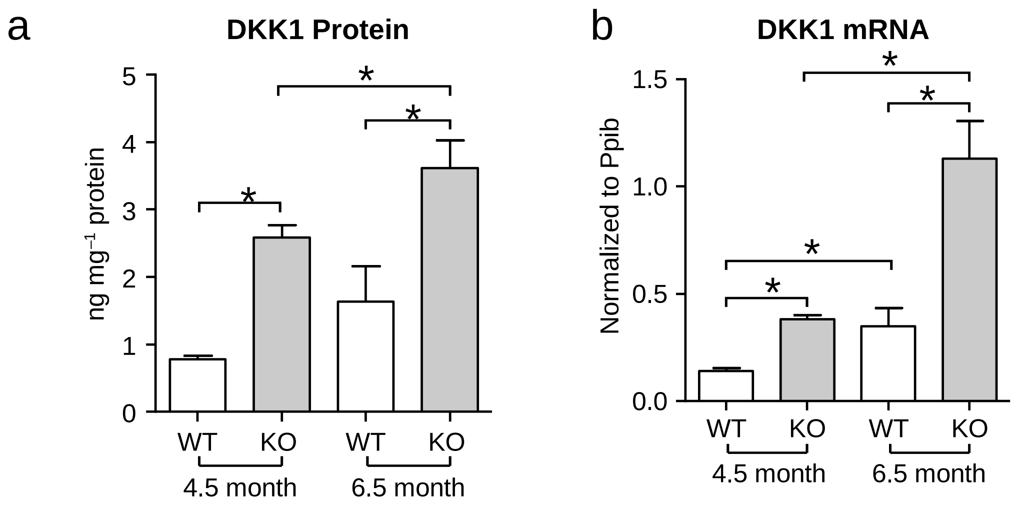

Supplementary Figure 1 DKK1 was elevated in sclerostin-inhibited mice. (**a**) DKK1 protein and (**b**) DKK1 mRNA were assessed in the tibiae of wild-type (WT) and SOST knockout (KO) mice (n = 10/group). Data are presented as Mean ± SE; *$P < 0.05$ as indicated by ANOVA + Tukey's *post-hoc* test.

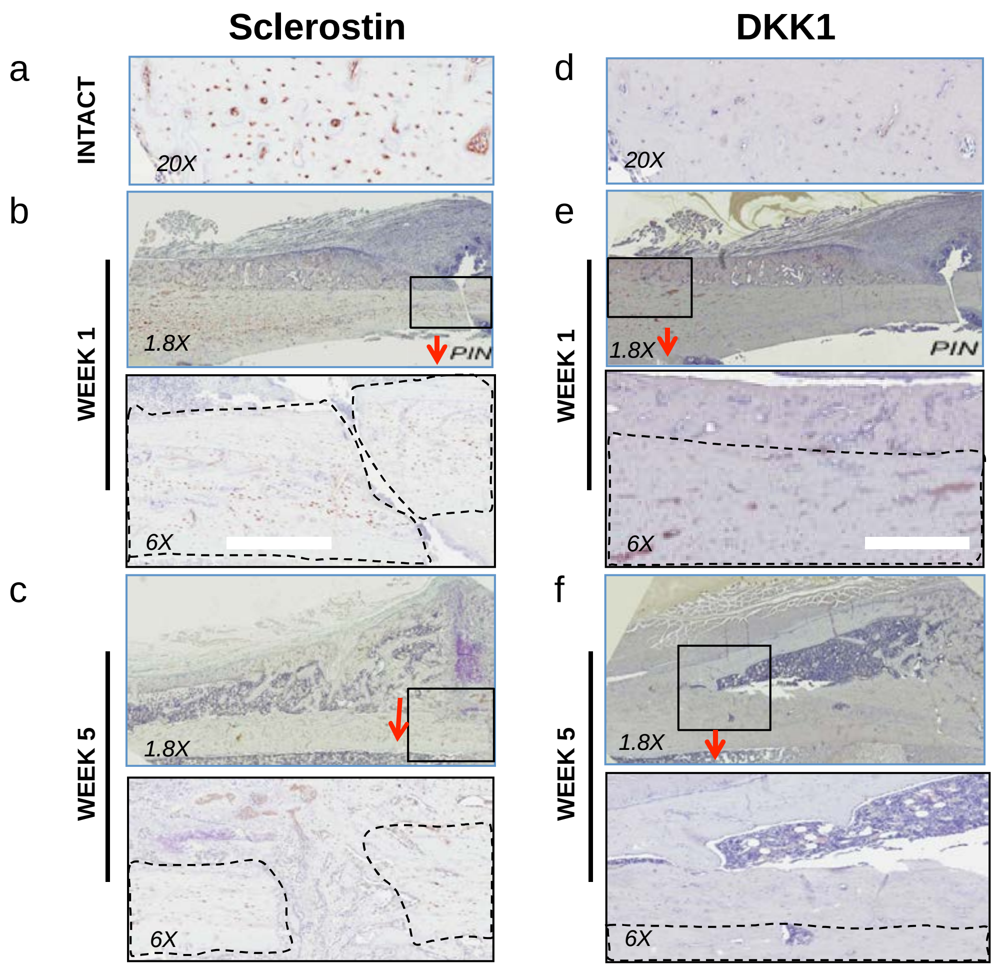

Supplementary Figure 2 Expression of sclerostin and DKK1 in rats with femoral closed fractures and intact contralateral femurs was monitored by immunohistochemistry in the cortical and callus regions over time at week 1 and week 5 post-fracture. Expression of sclerostin and DKK1 in the intact cortex is shown in (**a**) and (**d**). Expression of sclerostin and DKK1 at low and high magnification is shown in regions proximal and distal to the fracture site (boxed regions) at week 1 post-fracture (**b** and **e**, upper and lower panels and week 5 post-fracture (**c** and **f**, upper and lower panels). The position of the intramedullary pin is indicated and arrows point to magnified boxed regions of interest. White scale bars represent 500 μm.

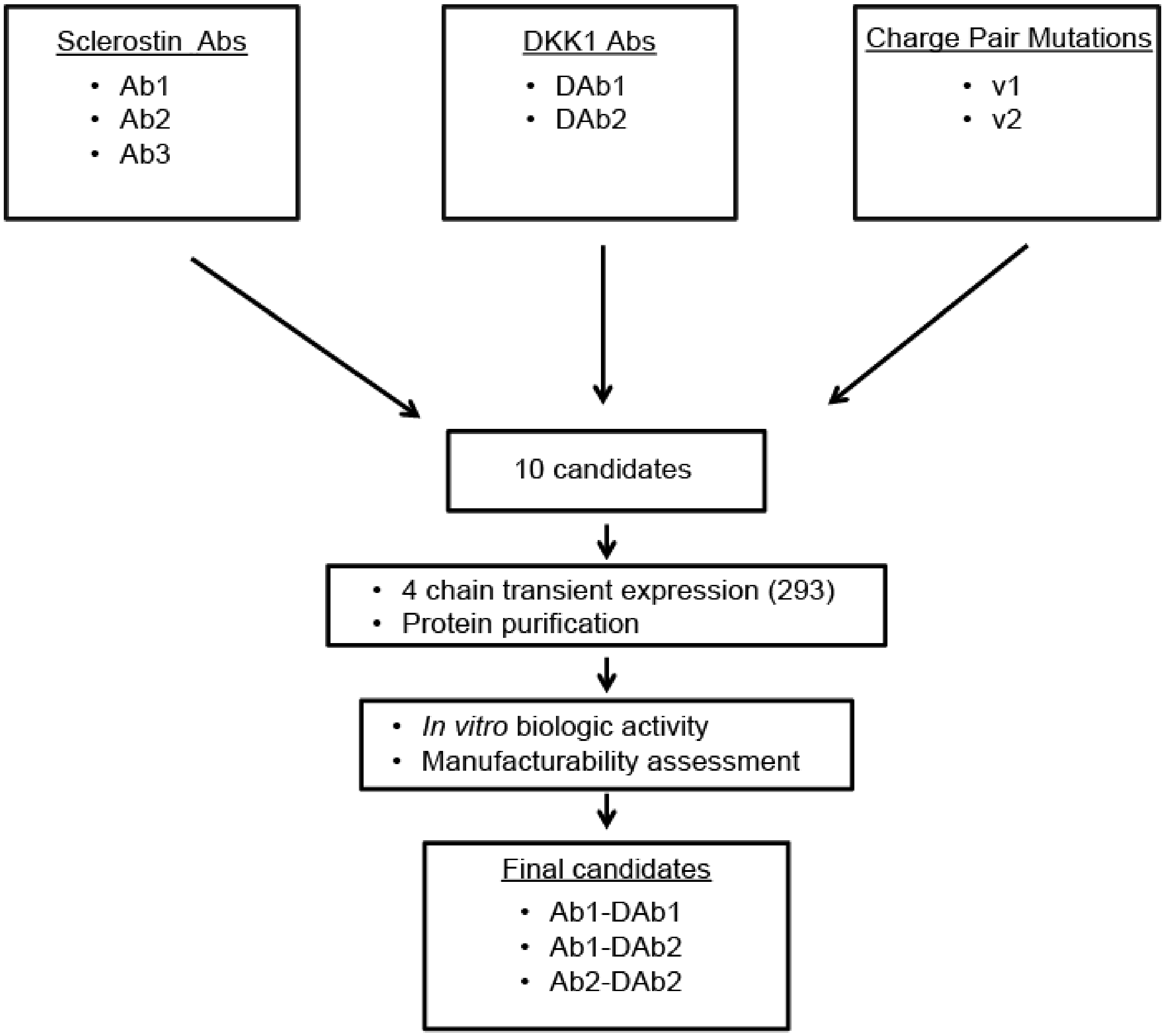

Supplementary Figure 3 Flow chart showing criteria for selection of lead Hetero-DS molecules. The Hetero-DS version 1 (v1) is shown in Figure 3a. Version 2 (v2) has an additional charge pair mutation at the VL-VH interface.

a

Antibody	Transient Expression Titers (mg L^{-1})
SAb	87
DAb	150
Hetero-DS	218

b

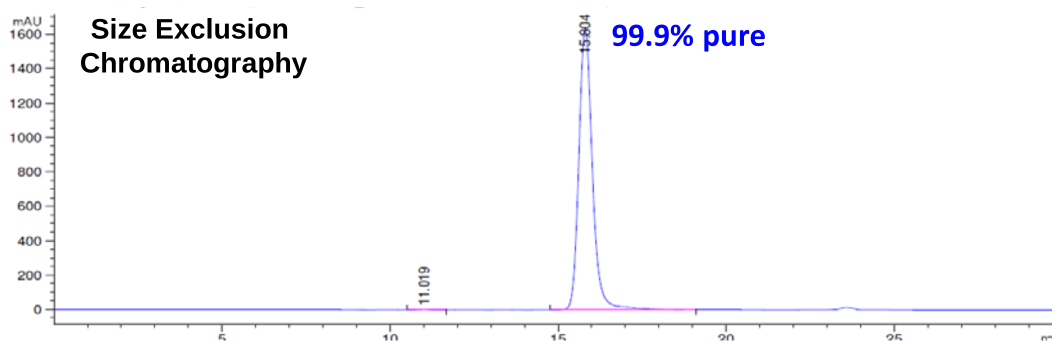

c

d

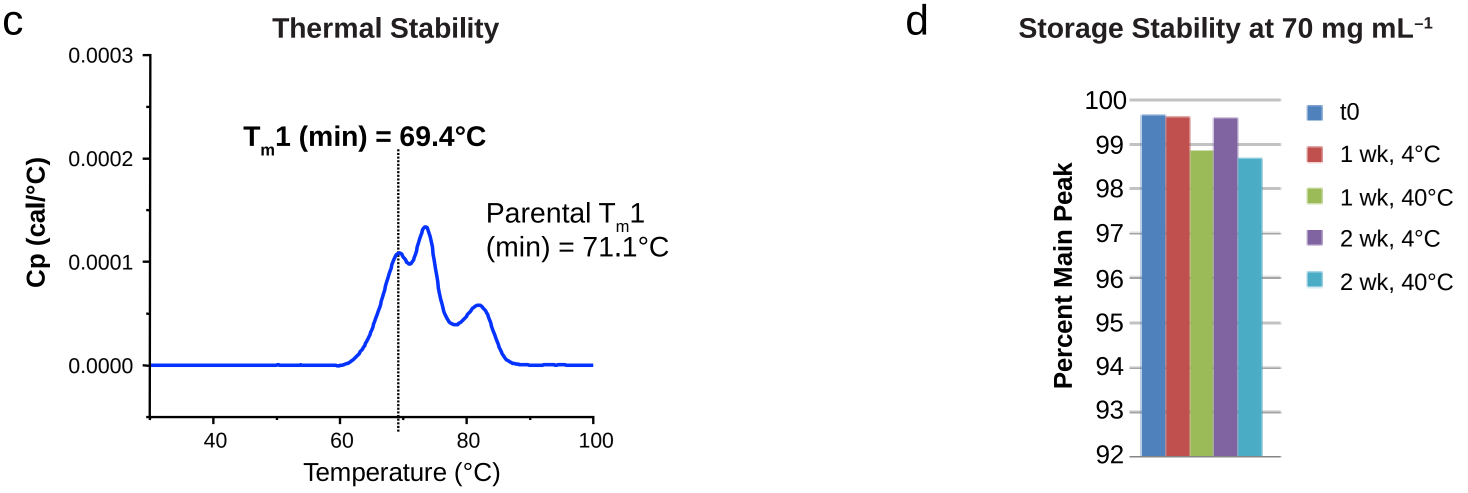

Supplementary Figure 4 Expression levels and biophysical properties of a lead Hetero-DS candidate. (**a**) Comparison of parental monospecific and engineered hetero-DS bispecific antibody transient expression levels. (**b**) Purity as measured by SEC during production. (**c**) Differential Scanning Calorimetry (DSC) profile. The lowest thermal transition (Tm1) for the Hetero-DS is 69.4°C compared to 71.1°C for the parental molecules. (**d**) Percent main peak as measured by Size Exclusion Chromatography (SEC) upon storage at high concentration (70 mg mL^{-1}) for up to 2 weeks at a temperature of 4°C and 40°C.

a

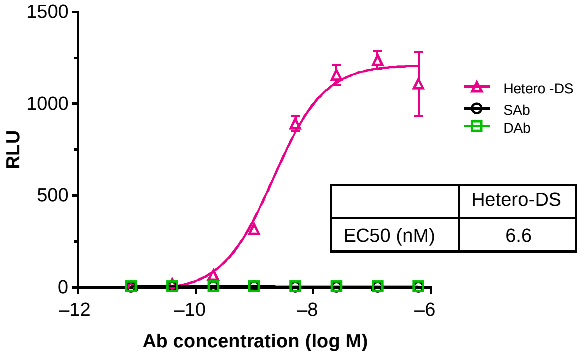

b

Antibody	MC3T3 Topflash Scl/DKK1 (nM)	KinExA huScl/ mScl (pM)	KinExA huDKK1/ mDKK1 (pM)	Dual capture ELISA EC50 (nM)	Scl/LRP6 AlphaScreen IC50 (nM)	DKK1/LRP6 AlphaScreen IC50 (nM)	AUC (hr*nM) IV 5 mg kg $^{-1}$	Rat PK t1/2 (hr)
Hetero-DS	14/1.8	42.2/4	702/1060	6.6	1.59	1.79	35,041	109
DAb	1.0	N/A	280.8/412	N/A	N/A	0.154	185,904	356
SAb	8	35.8/8	N/A	N/A	0.59	N/A	29,767	65.8

c

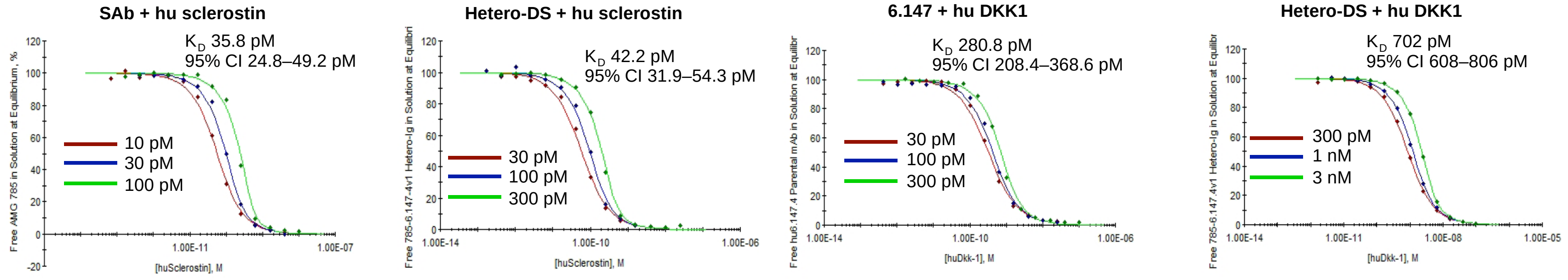

Supplementary Figure 5 Hetero-DS attributes. (**a**) MC3T3E1 Topflash Wnt reporter assay comparing the *in vitro* biologic activity of Hetero-DS, SAb and DAb in the presence of both Dkk1 and Sclerostin (*n* = 3). Data are presented as Mean ± SEM. (**b**) Table comparing Hetero-DS, SAb and DAb in an i) *in vitro* MC3T3 Topflash reporter assay in the presence of DKK-1 or sclerostin, ii) KinExA-determined binding activity to DKK-1 or sclerostin, iii) dual antigen binding enzyme-linked immunosorbent assay (ELISA) showing binding to both sclerostin and DKK-1 (*n* = 3), iv) LRP6/DKK1 and LRP6/sclerostin AlphaScreen assays (n=3), and v) pharmacokinetic (Rat PK) properties (*n*=3/group). Data are presented as Mean ± SEM. (**c**) KinExA-determined binding of Hetero-DS and parental antibodies to DKK-1 or sclerostin (*n*=3).

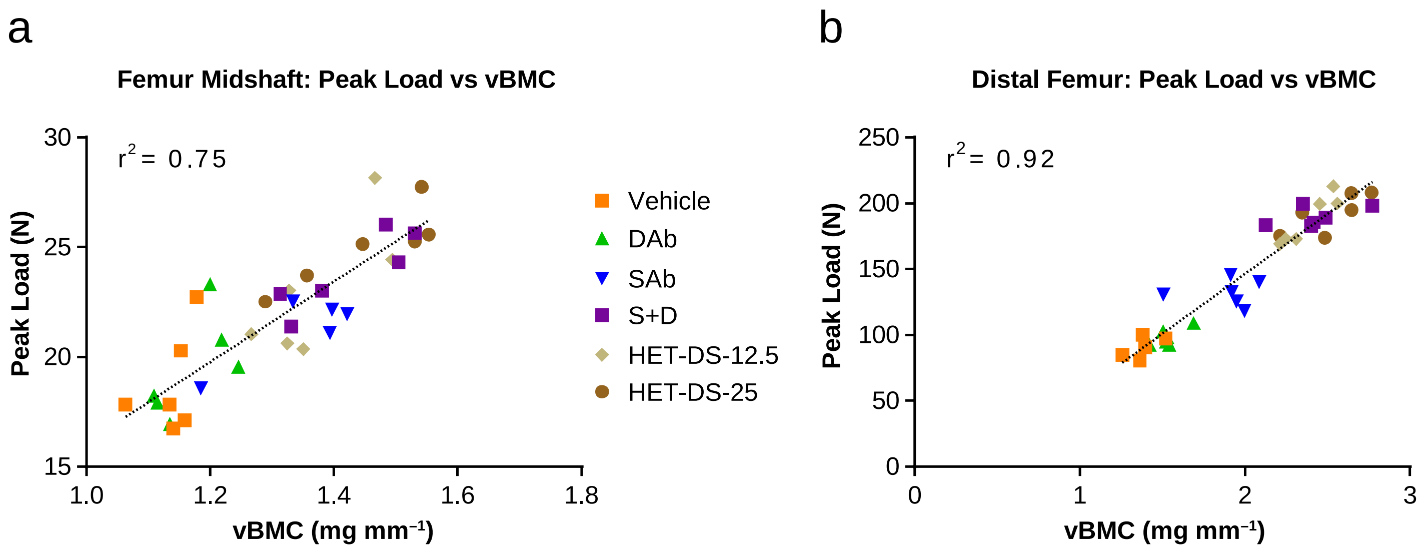

Supplementary Figure 6 Bone mass - bone strength regressions in mice. Ten-week-old male B6D2F1 mice were injected subcutaneously twice weekly with Vehicle, sclerostin antibody (Scl-Ab; 12.5 mg kg^{-1}), DKK1 antibody (DKK1-Ab; 12.5 mg kg^{-1}), Hetero-DS (12.5 and 25 mg kg^{-1}), or Scl-Ab + DKK1-Ab (S+D; 12.5 mg kg^{-1} each) for 3 weeks (n = 6/group). At the femur midshaft, volumetric bone mineral content (vBMC) was determined by micro-computed tomography (microCT) and peak load was determined by strength testing. Regressions and coefficients of variation between these parameters are shown at (**a**) the femur midshaft and (**b**) the distal femur across all treatment groups.

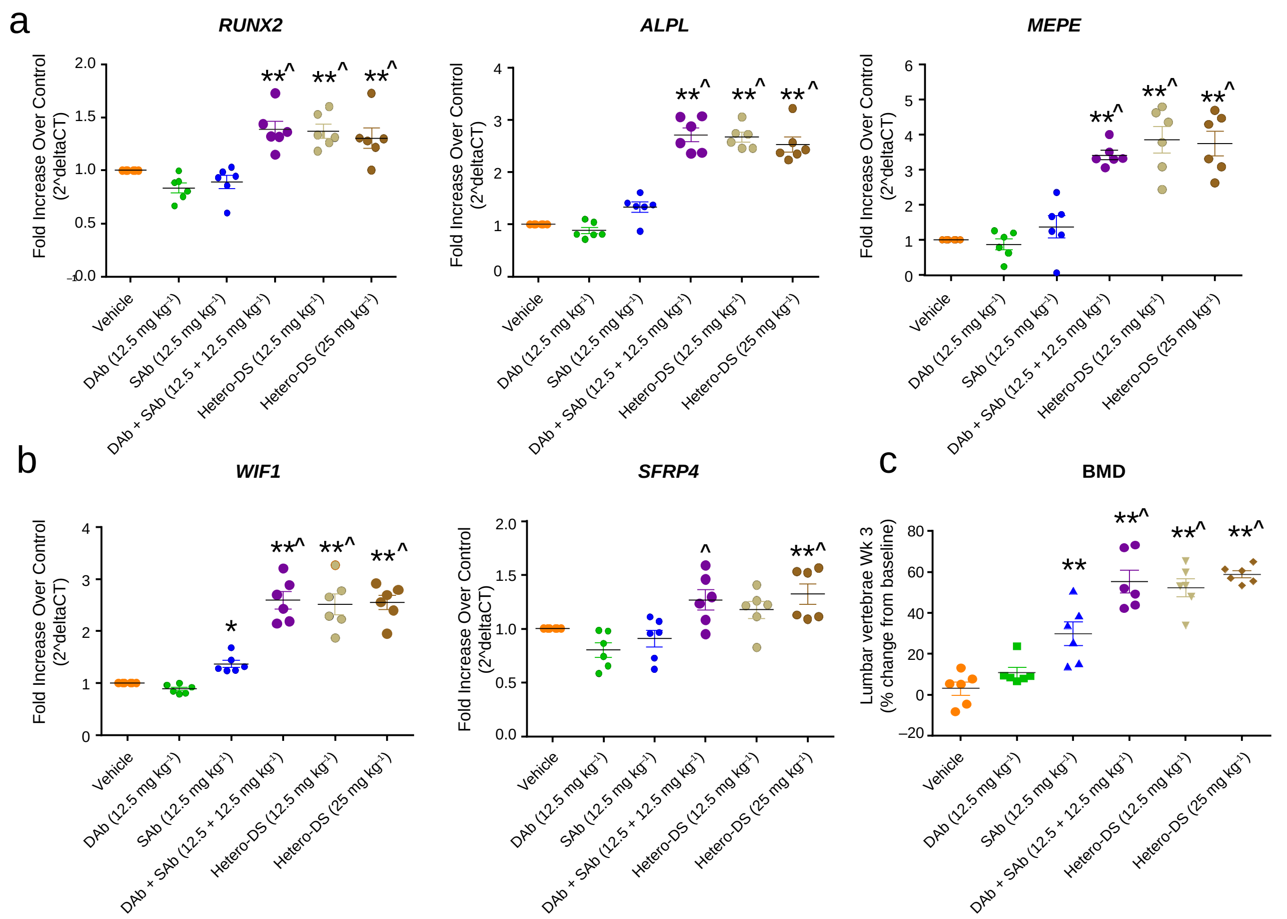

Supplementary Figure 7 Analysis of osteogenic gene expression in mice treated with monospecific, bispecific and combination therapy. *P < 0.05 vs Vehicle, **P < 0.01 vs Vehicle, ^P < 0.05 vs SAb; One-way ANOVA, Tukey's *post hoc* test. (**a**) Taqman gene expression analysis of osteogenic genes in the lumbar vertebrae of mice treated with Hetero-DS (n = 6 per group). Genes of interest shown were normalized to a housekeeping gene (*HPRT*). Data are presented as Mean ± SE. (**b**) Taqman gene expression analysis of Wnt antagonists *WIF1* and *SFRP4* following treatment with monotherapy and Hetero-DS in the lumbar vertebrae of young mice. (**c**) Analysis of bone mineral density (BMD) in the lumbar vertebrae of 10-week-old mice dosed for 3 weeks with Hetero-DS.

a

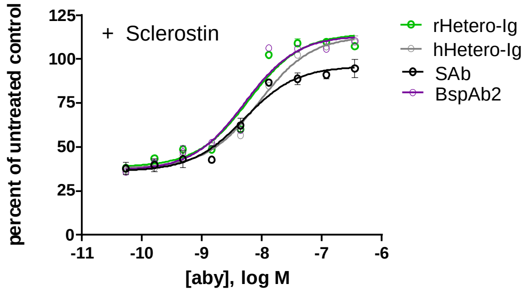

	rHetero-DS	hHetero-DS	BspAb2	SAb
EC50	6.153	9.1	5.27	5.16

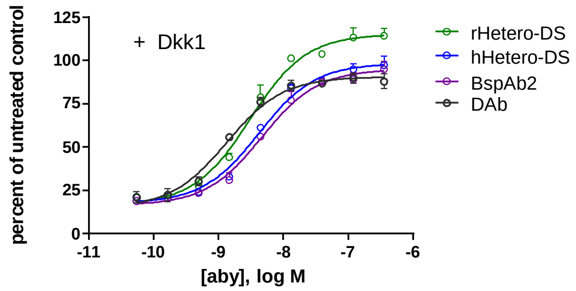

	rHetero-DS	hHetero-DS	BspAb2	DAb
EC50	3.04	4.2	4.67	1.35

b

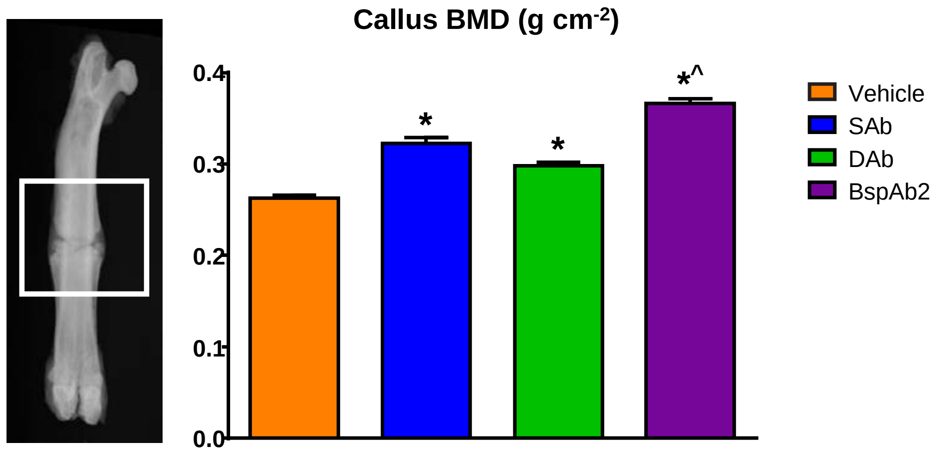

Supplementary Fig 8 Comparison of *in vitro* and *in vivo* biologic activity of bispecific constructs and monotherapies. (**a**) Bispecific constructs (rHetero-DS, hHetero-DS, BspAb2) and control parental antibodies (DAb and SAb) were compared in a MC3T3E1 Topflash Wnt reporter assay with the EC50s depicted in the insert tables. Data represent at least two experiments and are presented as Mean ± SEM. (**b**) Administration of BspAb2, DAb and SAb for 5 weeks in 3-month-old rats dose-dependently increased DXA BMD at the midshaft callus in a closed femur fracture model. Doses administered were 0.172 μmol kg^{-1}, 0.33 μmol kg^{-1} and 0.33 μmol kg^{-1} respectively. Data are from one experiment with 18 rats per treatment group. Data presented as Mean ± SEM; *$P < 0.05$ vs Vehicle and $^{\wedge}P < 0.05$ vs SAb and DAb by ANOVA + Tukey's *post hoc* test.

Supplementary Table 1 Bone histomorphometry in OVX rats

		Sham	OVX	Scl-Ab	DKK1-Ab	Combo
L2 Vertebra	**BV/TV (%)**	38.6 ± 1.0	27.8 ± 1.3^	57.4 ± 2.7*^	31.3 ± 1.2^	67.6 ± 1.5*^^{s}
	**ES/BS (%)**	2.41 ± 0.15	4.60 ± 0.36^	1.82 ± 0.27*	4.11 ± 0.37^	0.45 ± 0.11*^^{s}
	**Tb.Th (µm)**	86.4 ± 2.5	78.8 ± 3.7	185.6 ± 11.5*^	80.3 ± 3.2	244.0 ± 10.5*^^{s}
	**Tb.N (mm^{-1})**	4.48 ± 0.09	3.54 ± 0.12^	3.15 ± 0.15^	3.91 ± 0.09^	2.80 ± 0.10*^
	**MS/BS (%)**	37.1 ± 2.3	46.8 ± 2.2^	77.0 ± 2.3*^	38.8 ± 1.9	91.6 ± 1.5*^^{s}
	**MAR (µm day^{-1})**	0.83 ± 0.03	0.99 ± 0.02^	1.27 ± 0.03*^	1.09 ± 0.04^	1.40 ± 0.03*^^{s}
	**BFR/BS (µm^{3} µm^{-2} yr^{-1})**	0.30 ± 0.02	0.46 ± 0.02^	0.98 ± 0.04*^	0.33 ± 0.03	1.28 ± 0.04*^^{s}
Tibia	**Ct.B.Ar (mm^{2})**	3.64 ± 0.04	4.04 ± 0.07^	4.37 ± 0.11^	4.10 ± 0.09^	4.68 ± 0.14*^
	**Ct.Th (µm)**	518 ± 6	512 ± 8	603 ± 10*^	541 ± 13	637 ± 13*^
	**Ps.MS/BS (mm)**	34.9 ± 4.9	55.9 ± 4.8^	97.4 ± 2.4*^	68.7 ± 7.5^	97.3 ± 1.0*^
	**Ps.MAR (µm day^{-1})**	0.70 ± 0.08	0.85 ± 0.07	1.73 ± 0.17*^	0.98 ± 0.14	2.29 ± 0.19*^
	**Ps.BFR/BS (µm^{3} µm^{-2} yr^{-1})**	0.27 ± 0.06	0.49 ± 0.07	1.69 ± 0.17*^	0.73 ± 0.16	2.23 ± 0.19*^
	**Ec.MS/BS (%)**	14.4 ± 2.7	32.0 ± 3.8^	96.4 ± 1.3*^	43.6 ± 3.3*^	98.7 ± 0.8*^
	**Ec.MAR (µm day^{-1})**	0.46 ± 0.08	0.75 ± 0.04^	1.27 ± 0.06*^	0.78 ± 0.05^	1.57 ± 0.04*^^{s}
	**Ec.BFR/BS (µm^{3} µm^{-2} yr^{-1})**	0.12 ± 0.02	0.24 ± 0.04^	1.23 ± 0.06*^	0.33 ± 0.03^	1.55 ± 0.03*^^{s}
	**Ec.ES/BS (%)**	2.41 ± 0.62	8.12 ± 1.10^	0.00 ± 0.00*^	2.64 ± 0.52*	0.00 ± 0.00*^^{s}

Mean ± SE. ^*P* < 0.05 vs Sham, **P* < 0.05 vs OVX, ^{s}*P* < 0.05 vs Scl-Ab by ANOVA + Tukey's

Supplementary Table 2 Bone histomorphometry in mice treated with Hetero-DS

		Vehicle	DKK1-Ab	Scl-Ab	Hetero-DS 12.5	Hetero-DS 25	Combo
**Distal Femur**	**BV/TV (%)**	13.4 ± 0.7	18.6 ± 0.6	27.7 ± 1.6*	50.9 ± 2.7*^{S}	50.3 ± 2.4*^{S}	48.0 ± 1.7*^{S}
	**ES/BS (%)**	5.73 ± 0.74	4.88 ± 0.72	4.18 ± 0.60	1.13 ± 0.15*^{S}	0.68 ± 0.23*^{S}	1.11 ± 0.46*^{S}
	**Tb.Th (µm)**	31.0 ± 1.3	38.1 ± 1.2	56.4 ± 3.9*	93.3 ± 6.8*^{S}	94.6 ± 5.7*^{S}	94.2 ± 6.3*^{S}
	**Tb.N (mm^{-1})**	4.35 ± 0.24	4.90 ± 0.16	4.95 ± 0.22	5.50 ± 0.13*	5.35 ± 0.17*	5.18 ± 0.26
	**MS/BS (%)**	34.8 ± 6.1	31.4 ± 3.7	69.4 ± 1.7*	87.3 ± 1.4*^{S}	91.4 ± 2.1*^{S}	92.3 ± 2.3*^{S}
	**MAR (µm day^{-1})**	0.64 ± 0.04	0.66 ± 0.02	0.80 ± 0.02*	0.84 ± 0.02*	0.83 ± 0.03*	0.79 ± 0.05*
	**BFR/BS (µm^{3} µm^{-2} yr^{-1})**	80.0 ± 14.7	76.3 ± 10.2	203.2 ± 7.7*	268.6 ± 10.7*^{S}	275.9 ± 8.7*^{S}	266.5 ± 14.0*^{S}
**Femur Diaphysis**	**Ct.B.Ar (mm^{2})**	0.77 ± 0.02	0.84 ± 0.02	0.98 ± 0.02*	0.91 ± 0.03*	0.98 ± 0.02*	1.01 ± 0.05*
	**Ct.Th (µm)**	187.8 ± 7.1	210.6 ± 2.3	245.2 ± 3.7*	231.7 ± 5.3*	240.8 ± 2.7*	247.5 ± 5.8*
	**Ps.Pm (mm)**	4.90 ± 0.04	4.79 ± 0.08	4.96 ± 0.10	4.83 ± 0.11	5.00 ± 0.09	5.09 ± 0.22
	**Ec.Pm (mm)**	3.37 ± 0.11	3.20 ± 0.08	2.99 ± 0.05*	3.06 ± 0.06	3.16 ± 0.08	3.02 ± 0.08*
	**Ps.MS/BS (%)**	24.4 ± 3.2	24.0 ± 2.5	65.0 ± 4.6*	59.8 ± 4.6*	86.4 ± 4.2*	75.4 ± 8.8*
	**Ps.MAR (µm day^{-1})**	0.48 ± 0.04	0.45 ± 0.02	0.80 ± 0.03*	0.81 ± 0.05*	0.94 ± 0.03*	0.83 ± 0.07*
	**Ps.BFR/BS (µm^{3} µm^{-2} yr^{-1})**	44.2 ± 8.6	39.6 ± 4.7	190.8 ± 15.6*	178.3 ± 19.8*	298.7 ± 21.3*^{S}	233.7 ± 36.9*
	**Ec.MS/BS (%)**	52.6 ± 11.0	70.9 ± 9.2	99.6 ± 0.3*	97.1 ± 1.0*	97.6 ± 0.9*	97.4 ± 0.5*
	**Ec.MAR (µm day^{-1})**	0.56 ± 0.05	0.55 ± 0.03	0.73 ± 0.05	0.92 ± 0.03*	0.92 ± 0.04*^{S}	0.95 ± 0.06*^{S}
	**Ec.BFR/BS (µm^{3} µm^{-2} yr^{-1})**	114.5 ± 30.7	142.5 ± 18.7	268.9 ± 17.9*	324.0 ± 7.8*	328.6 ± 12.8*	336.7 ± 18.9*
	**Ec.ES/BS (%)**	9.77 ± 0.86	4.90 ± 1.13*	0.00 ± 0.00*	0.85 ± 0.73*	0.00 ± 0.00*	0.40 ± 0.27*

Mean ± SE. *$P < 0.05$ vs Vehicle, $^{s}P < 0.05$ vs Scl-Ab by ANOVA + Tukey's
